# IL-17 stimulates erythropoiesis *in vivo* by amplifying the response of erythroid progenitors to erythropoietin

**DOI:** 10.1371/journal.pbio.3003462

**Published:** 2025-12-11

**Authors:** Qiu C. Wu, Aishwarya Swaminathan, Ashley Winward, Logan Lalonde, Yung Hwang, Noah Littman, Merav Socolovsky, Allon M. Klein

**Affiliations:** 1 Department of Systems Biology, Harvard Medical School, Boston, Massachusetts, United States of America; 2 Department of Molecular, Cell and Cancer Biology, UMass Chan Medical School, Worcester, Massachusetts, United States of America; National Cancer Institute, UNITED STATES OF AMERICA

## Abstract

Red blood cell production is regulated by erythropoietin (Epo), maintaining tissue oxygen tension in the steady state and in response to stress. To date, only a handful of factors other than Epo are known to stimulate erythropoiesis, limiting therapeutic options. We recently found that IL-17, a pleiotropic pro-inflammatory cytokine, interacts synergistically with Epo to increase formation of erythroid colonies *in vitro*. Here, we administered IL-17 to mice to determine whether it accelerates erythropoiesis *in vivo*. We found that while IL-17 alone had little effect on erythroid and other hematopoietic lineages, combined treatment with both IL-17 and Epo generated a specific and strong synergistic response in erythroid progenitors that significantly increased erythropoietic rate. IL-17 administration also accelerated the erythropoietic response of mice to hypoxia. Single-cell transcriptomic analysis showed that IL-17 acts by sensitizing erythroid progenitors to Epo, rather than through a distinct transcriptional response. Using a dynamical model, we propose that this mechanism optimizes conflicting requirements in the regulation of erythropoiesis, balancing the need for low-cost maintenance of the steady state, with a sufficiently fast stress response. Further, our findings suggest a potentially broadly applicable mechanism whereby pleiotropic cytokines are able to exert lineage-specific effects when their actions are dependent on synergism with lineage-specific factors.

## Abstract 2

Erythropoietin (Epo) can be an effective anemia treatment, but is ineffective or contraindicated in anemia associated with cancer or chronic disease. This study identifies the pleiotropic cytokine IL-17A as a specific stimulator of erythropoiesis *in vivo* that acts by amplifying the response of erythroid progenitors to Epo.

## Introduction

Erythropoiesis is under tight feedback control to maintain optimal tissue oxygen tension. Red cells, which carry oxygen from lungs to tissues, constitute 84% of all body cells [1] and turn over every 100 days [2,3]. Erythropoiesis therefore consumes substantial resources and is vulnerable to stresses such as nutritional deficiencies, inflammation, and cancer, which can lead to anemia. In addition to optimizing available resources and maintaining red cell number and tissue oxygen tension within a narrow range, feedback control of erythropoiesis must have the capacity for mounting a rapid and quantitatively appropriate stress response when tissue oxygen tension is threatened, as in blood loss or hypoxia [2–4]. In response to these varying stresses, erythropoietic rate increases up to 10-fold within a matter of hours. The circuits responsible for this complex regulation are only partly understood.

**Fig 1:**
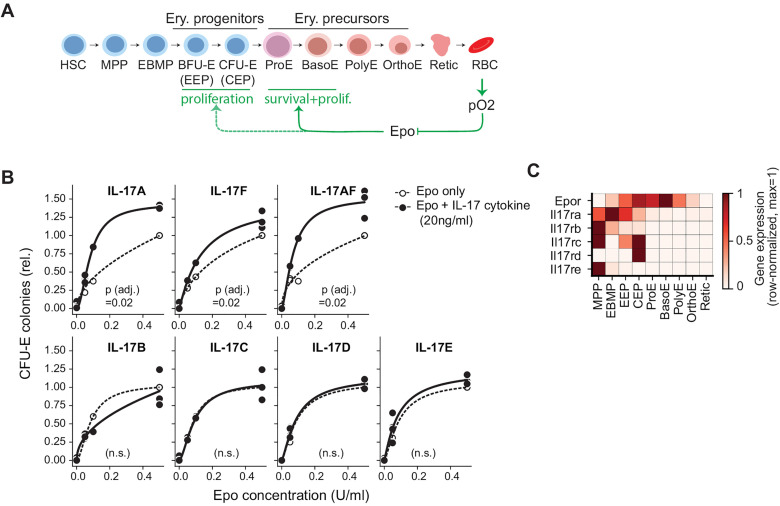
IL-17 ligands with erythropoietic activity *in vitro.* **(A)** The erythropoietic negative feedback cell circuit. Abbreviations: HSC, hematopoietic stem cell; MPP, multipotent progenitor; EBMP, erythroid/basophil-mast cell/megakaryocytic progenitor; BFU-E/EEP, erythroid burst-forming unit or early erythroid progenitor; CFU-E/CEP, erythroid colony-forming unit or committed erythroid progenitor; ProE, pro-erythroblast; Baso/Poly/OrthoE, basophilic, poly-, and ortho-chromatic erythroblasts; Retic, reticulocyte; RBC, red blood cell. **(B)** CFU-E colony formation assays with IL-17 cytokines. Mouse bone marrow cells were plated in the presence of Epo at the indicated concentrations, and either in the presence or absence of IL-17 ligands. CFU-E colonies were scored at 72 hours. Data are pooled from *n* = 3 independent experiments for IL-17A, F, AF, D, E, and *n* = 2 for IL-17B, C with 3 to 4 technical replicates per experiment. Lines show fits to a Hill function. *p*_adj_ values are Wilcoxon signed-rank test across all Epo concentrations, with Benjamini–Hochberg (BH) correction across all IL-17 ligands. Data is expressed relative to the arithmetic mean for all replicates of the CFU-E colony number at Epo = 0.5 U/ml (without added IL-17 cytokines) in each experiment. Data for this figure panel are in S1 Table. (C) Expression of Epor and IL-17 receptor mRNAs in erythroid progenitors. Data derived from single-cell RNA sequencing from this paper, further elaborated in Fig 4. Values show row-normalized gene expression, log(1 + CP10K)/log(1 + CP10K_max_). Data for this figure panel are in S10 Table.

Erythropoiesis consists of two principal phases [5,6] (Fig 1A): an early progenitor phase followed by erythroid terminal differentiation (ETD). In the early progenitor phase, hematopoietic stem cells and multipotent progenitors (MPPs) differentiate into erythroid progenitors known as the burst-forming and colony-forming erythroid units (BFU-E and CFU-E), which undergo transit-amplifying divisions. A transcriptional switch then initiates ETD [5,7], where erythroid precursors comprising proerythroblasts (ProE), early and late erythroblasts, undergo 3–5 maturational cell divisions and enucleate into nascent red cells (reticulocytes). Homeostatic control is established through a lineage-specific and essential feedback mediator, the cytokine hormone erythropoietin (Epo). Epo is secreted by the adult kidney; its production increases in response to decreasing tissue oxygen tension [8,9]; and it in turn increases erythropoiesis by promoting survival [10–12] and cycling [13] of ProE and early erythroblasts in ETD in a dose-dependent manner. Thus, Epo mediates a closed homeostatic feedback loop (Fig 1A).

Epo also regulates the early progenitor phase prior to ETD, but its regulatory function in these cells is not well explored. MPPs, BFU-E, and early CFU-E express the Epo receptor (EpoR) [5] but do not require Epo for survival [14] and, *in vitro*, are less sensitive to Epo than ETD precursors [15–17]. In addition to Epo, the early progenitor compartment is also a target for several other factors with pan-hematopoietic or multi-lineage effects such as stem-cell factor (SCF), BMP4, and glucocorticoids that have established roles in the erythroid stress response [18–21]. Further, our recent single-cell transcriptomic analysis revealed additional growth factor and cytokine receptors expressed in erythroid progenitors that were not previously known to regulate erythropoiesis [5]. One such receptor is IL-17RA, whose ligand, IL-17A, is a pro-inflammatory cytokine produced by T cells and other immune cells [22,23] with pleiotropic tissue targets [24,25]. Using colony-formation assays *in vitro*, we found that IL-17A enhances the response of both human and mouse CFU-E to Epo [5]. Whether IL-17A stimulates erythropoiesis *in vivo*, and, given its pleiotropic nature, whether it can exert such an effect without perturbation to other lineages, has not yet been tested.

Here, we addressed these questions by administering IL-17A to wild-type and IL-17RA-mutant mice in normoxia and hypoxia, and characterizing their hematopoietic and erythropoietic responses *in vivo* using flow-cytometric and single-cell RNA sequencing (scRNA-seq) approaches. We found that while IL-17A administered alone generates little response, it has a striking synergistic interaction with Epo that augments and accelerates the response to hypoxic stress. This response is specific to erythropoiesis with little effect on other hematopoietic lineages. Mechanistically, synergism is attained through an IL-17A-mediated increase in the sensitivity of erythroid progenitors to Epo. A dynamical model suggests that this is an efficient mechanism for tuning Epo-mediated feedback control. Our work establishes IL-17A as a novel erythropoiesis-stimulating factor *in vivo*, with potential therapeutic roles in anemia. Further, it offers a template for understanding its action in other cell lineages, and suggests how broadly acting environmental cues and pleiotropic factors regulate progenitors to achieve lineage-specific responses.

## Results

### IL-17A, IL-17AF, and IL-17F stimulate erythroid colony formation

We recently found that *Il17ra*, encoding the IL-17 receptor A chain (IL17RA), is expressed by early hematopoietic and erythroid progenitors [5], and that its ligand, IL-17A, markedly potentiates formation of CFU-E colonies from adult mouse or human bone marrow, with saturable kinetics and an EC_50_ of ~60 pM. This effect is lost in cells from *Il17ra*^*−/−*^ mice [5]. IL-17RA is a common receptor chain in several heteromeric receptor complexes, each of which binds distinct members of the IL-17 cytokine family [23,24]. Here, we examined the remaining six IL-17 family ligands for potential erythropoietic activity using CFU-E colony-formation assays (Fig 1B). We found that, like IL-17A, the homodimeric IL-17F, and the heterodimeric IL-17AF, also enhanced CFU-E colony formation in response to Epo, and that none had an effect in the absence of Epo. By contrast, the remaining homodimeric IL-17 ligands, IL-17B, C, D, and E, had no effect. IL-17A, F and AF (each at 20 ng/ml) increased the response to Epo (0.5 U/ml) by 39% ± 1.4%, 21% ± 0.7%, and 46% ± 1.1%, respectively (mean ± SEM, adjusted *p* value (*p*_adj_) = 0.02 for each cytokine, Wilcoxon signed-rank test paired by Epo concentration, with Benjamini–Hochberg (BH) correction across cytokines). IL-17A, F, and AF are the three known ligands for the IL-17RA/IL-17RC receptor complex [23,26], suggesting that this is the functional IL-17 receptor in erythroid progenitors. Both these receptor chains are expressed in erythroid progenitors (Fig 1C).

As a technical note, the efficacy of IL-17A, F, and AF in CFU-E assays was sensitive to the age of the lyophilized proteins and to the length of storage after reconstitution (S1A Fig). For all subsequent experiments, we used cytokines with lyophilization age less than 1 year to minimize the source of variation. The non-responsiveness to IL-17D and E could not; however, be explained by cytokine age. We focused our *in vivo* experiments on IL-17A, since there are no commercially available recombinant IL-17F or IL-17AF preparations from mammalian-cell systems.

### IL-17A and Epo synergize *in vivo* to increase erythropoietic rate

**Fig 2:**
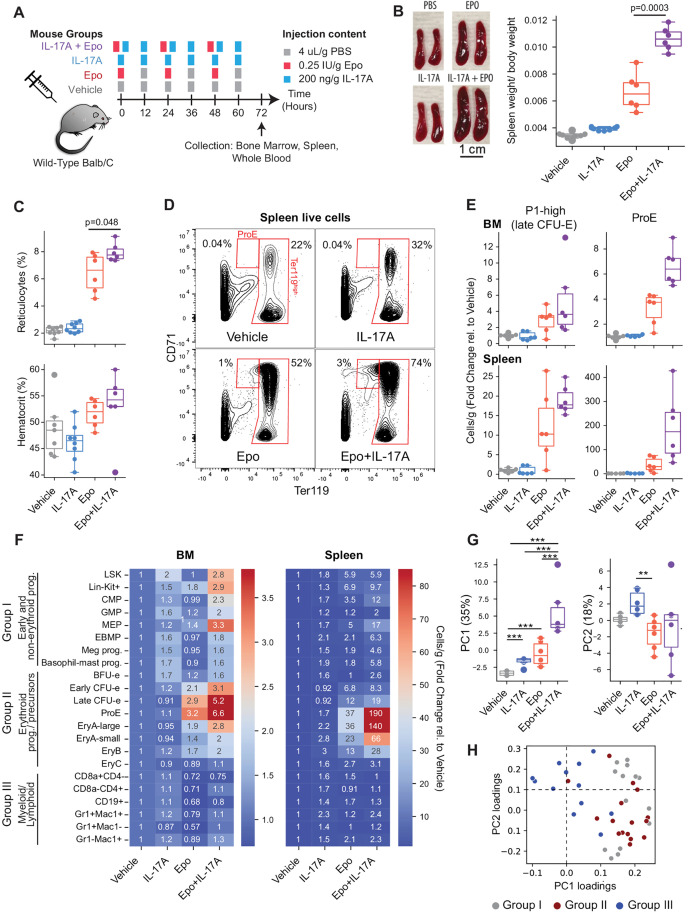
IL-17A synergizes with Epo to increase erythropoietic rate *in vivo.* **(A)** Experimental design to evaluate IL-17A erythropoietic stimulation *in vivo* for data in panels B to G. The data were pooled from 3 independent similar experiments, with a total of 6 to 8 mice per treatment. FACS gate definitions and full data are in S2, S3 and S4 Figs. **(B)** Representative images of freshly-isolated spleens at 72 hours and spleen to body weight ratios. *p*-values are from two-tailed *t*-tests. Here and in all subsequent boxplots, data points correspond to individual mice, box shows medians and the central quartiles (25th to 75th percentiles), and whiskers extend by 1.5 times the inter-quartile range. Data for this figure are in S2 Table. **(C)** Changes in blood reticulocyte fraction (CD71^+^ Draq5^-^ blood cells) and hematocrit. **(D)** Representative flow-cytometric 2D contour plots of Ter119 (erythroid-specific) and CD71 expression, identifying ProE and later erythroid precursors (Ter119^high^ gate). Erythroid precursors are sub-divided into increasingly differentiated erythroblast gates EryA, B, and C as in [29], shown in S2B Fig. **(E)** Summary data of bone marrow (BM) and spleen cells labeled with either a 10-color stain antibody-panel to identify CFU-E [5,30] (S3A Fig) or with the CD71/Ter119 panel to identify ProE (panel D). **(F)** Heat map summarizing the fold-change in the mean cell number (cells/g body weight) of *n* = 6 mice per condition relative to vehicle-treated mice, for each flow-cytometric subset in bone marrow (BM) and spleen. Full data for individual subset/mice is given in panel E, S2, S3 and S4 Figs, and S2 Table. Groups I–III indicate functionally distinct progenitor subsets. **(G)** Principal component (PC) analysis of the entire dataset, treating the 22 bone marrow and 22 spleen subsets as independent features of the data, for all mice in the 4 treatment groups. Individual datapoints correspond to individual mice. The y-axes are PC1 and PC2, which explain 35% and 18% of the between-mouse variance. Statistical significance was assessed with Kruskal-Wallis *H*-test across all treatments (one-way ANOVA on ranks; *p* = 0.0002 for PC1, *p* = 0.0147 for PC2) and a Conover test was used for post-hoc pairwise comparisons with Benjamini–Yekutieli (BY) correction: ***, *p*_adj_ < 10^−3^; **, *p*_adj_ < 10^−2^. **(H)** Loadings of PC1 and PC2. Each dot corresponds to FACS subset in the dataset. FACS subsets are colored by the groups defined in panel F. PC1 is enriched for early hematopoietic progenitors (Group I) and erythroid progenitors and precursors (Group II; *p* = 1.45 × 10^−6^, Wilcoxon rank sum test comparing PC1 loadings for Groups I and II vs. Group III). PC2 is enriched for myeloid and lymphoid subsets (Group III, *p* = 0.029). See S2E and S2F Fig for the identity and tissue of each of the FACS subsets. Data for all figure panels are in S2 Table.

We next examined whether IL-17A promotes erythropoiesis *in vivo*. We established an optimal dosing regimen by undertaking pharmacokinetic analysis of IL-17A (S2A Fig, [27]). We then treated 8–12-week-old Balb/c mice with either vehicle (phosphate-buffered saline, PBS), IL-17A (200 ng/g every 12 hours), Epo (0.25 U/g every 24 hours), or both Epo and IL-17A (*n* = 6–8 mice per treatment group, pooled from 3 independent experiments) (Fig 2A). Mice were sacrificed at 72 h, and blood, bone marrow, and spleen were collected for a comprehensive analysis of hematopoietic progenitors and mature blood cells (Figs 2, S2, S3 and S4).

The mouse spleen is the principal extra-medullary site of expansion of hematopoietic and erythropoietic tissue in response to stress signals. We found that administration of IL-17A by itself had little effect on spleen size, and that, as expected, Epo increased spleen size by 2-fold. Remarkably, the combined Epo and IL-17A treatment increased spleen size by nearly 3-fold [Fig 2B; spleen to body weight ratio for vehicle, 0.34 ± 0.008% (mean ± SEM); for Epo, 0.67 ± 0.06%; and for Epo + IL-17, 1.0 ± 0.03%; the difference between Epo and Epo + IL-17A is significant at *p* = 0.0003, two-tailed *t* test].

Spleen enlargement suggested an increase in erythropoietic rate, which was confirmed when we examined blood reticulocytes (which are reflective of erythropoietic rate). Reticulocyte numbers were increased by Epo as expected, and further significantly increased, compared with Epo alone, by the combined Epo + IL-17A treatment (Fig 2C, *p* = 0.048). Given that the hematocrit was unaltered in any of the treatment groups (Fig 2C), the increase in reticulocytes was not a compensatory response to anemia/hypoxia. Of note, 72 hours of erythropoietic stimulation is too short a period for an increase in erythropoietic rate to result in a significant increase in hematocrit.

We proceeded with comprehensive examination of early hematopoietic, erythroid, myeloid and lymphoid cells in the bone marrow and spleen (Figs 2D–2H, S2–S4; S2 Table) using flow cytometry [28,29], including a recent flow-cytometric strategy that identifies highly pure BFU-E, CFU-E and erythroid/megakaryocytic/basophil-mast cell progenitors (EBMPs) (S3A Fig, the “10 color stain”) [5,30]. This analysis showed that administration of IL-17A alone has little or modest (<2 fold) effects on hematopoietic progenitors, including minimal effects on myeloid and lymphoid cells (Figs 2F, S2C, and S2D). In combination with Epo, however, IL-17A markedly increased the absolute numbers of BFU-E, CFU-E, erythroid precursors and early hematopoietic progenitors that contribute to the erythroid trajectory (EBMP, MEP); the expansion in these populations was well above that observed in response to Epo alone (Figs 2D–2F, S2B, and S3C–S3E). Peak response to the combined Epo + IL-17A treatment was in CFU-E, ProE, and EryA precursors; for example, spleen ProE increased 37 ± 12-fold in response to Epo alone, and 193 ± 58-fold in response to the combined Epo + IL-17A treatment (Figs 2D–2F and S2B).

Because changes in different cell sub-populations are not independent of one another, we carried out a statistical analysis of changes in progenitors after first binning them into three principal groups: early hematopoietic progenitors (group I); erythroid progenitors-and-precursors (group II); and myeloid-and-lymphoid progenitors (group III). We found a statistically significant increase in the absolute number of progenitors in groups I and II when comparing the combined treatment of Epo + IL-17A with treatment with Epo alone (*p*_adj_ = 0.017 in bone marrow, *p*_adj_ = 0.03 in spleen, Wilcoxon signed-rank test paired by FACS gate, with BH correction across comparisons). There was no significant difference between the combined treatment and treatment with Epo alone on group III (myeloid and lymphoid progenitors, Figs 2F, S2C and S2D). These same changes can also be appreciated without prior grouping of progenitors by principal component analysis (PCA) of the 44 flow-cytometric bone marrow and spleen subsets in each of the four treatment conditions. The first principal component (PC) explains 35% of the variance between mice from all conditions (Fig 2G) and corresponds to the response by early hematopoietic and erythroid subsets (Groups I and II), as seen from its loading coefficients (Fig 2H). This PC is most affected by the combined Epo + IL17A treatment, less so by Epo treatment alone, and least by IL-17A alone (Fig 2G). By contrast, the second PC (which explains 18% of the variance in the dataset) is enriched for myeloid and lymphoid subsets, including GMP progenitors, CD4^+^ and Gr^+^/Mac1^+^ precursors, and is responsive to treatment with IL17-A alone (Figs 2G, 2H, S2E and S2F).

Together, these results show that IL-17A synergizes with Epo: it is an erythropoietic-stimulating factor *in vivo*, but only in the context of elevated Epo, as might be seen in erythropoietic stress.

In these experiments, we also examined the fraction of cells that are undergoing apoptosis in each flow cytometric subset, using Annexin V binding (S4D Fig). As established previously [11,29], Epo administration reduced erythroblast apoptosis. The combined Epo + IL-17A treatment had a quantitatively similar effect to Epo alone, suggesting that IL-17A does not exert its synergistic effect through the regulation of erythroblast apoptosis.

### IL-17A is dispensable for baseline erythropoiesis

**Fig 3:**
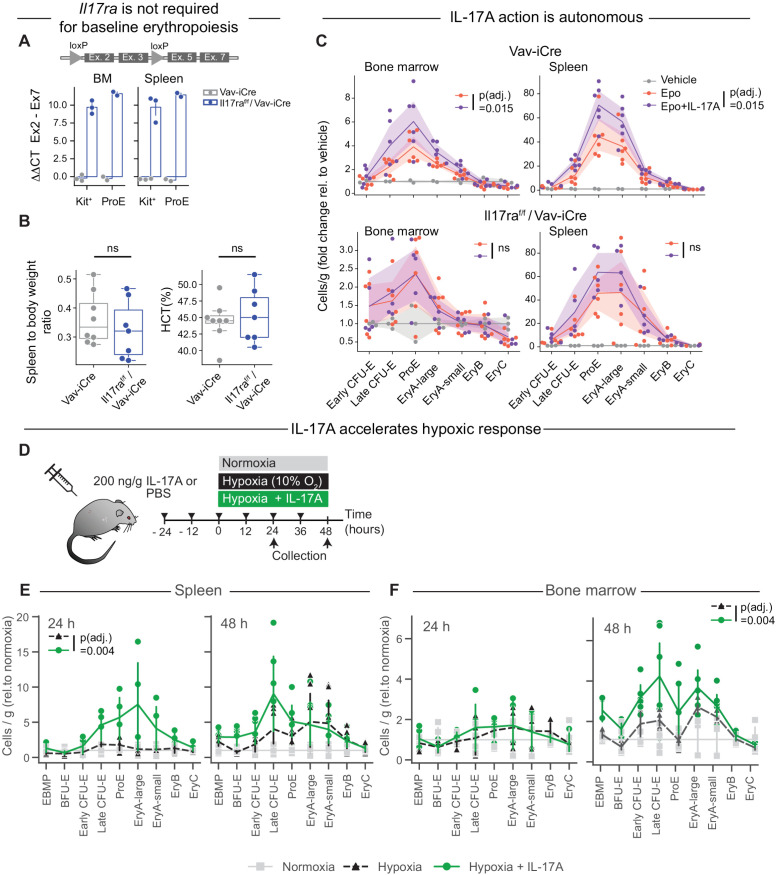
The erythropoietic effect of IL-17A is autonomous to hematopoietic cells and accelerates the response to hypoxic stress. **(A)** Deletion efficiency of *Il17ra* in the hematopoietic-specific Vav-iCre model, evaluated by genomic qPCR of the excised genomic locus (exons 2,3) normalized to an adjacent locus (exons 5,7). The cartoon of the *Il17ra* locus is not to scale. qPCR was performed on FACS-sorted Kit^+^ and ProE cells. Data points are independent biological replicates. Additional analysis confirming deletion using independent primers is in S5A Fig. Data for this figure panel are in S10 Table. **(B)** The IL-17RA receptor is not required for steady-state erythropoiesis. Spleen to body weight ratio and hematocrit in Il17ra^f/f^/ Vav-iCre mice and in control Vav-iCre mice. Analysis of hematopoietic subsets for these mice are shown in S5B Fig. Data for this figure panel are in S10 Table. **(C)** Erythroid subsets (fold change) at 72 hours following cytokine injections, in control Vav-iCre and Il17ra^f/f^/ Vav-iCre mice. Cytokine injections followed the schedule in Fig 2A. Data are pooled from 2 independent experiments, *n* = 6 mice per treatment. Color bands are 95% confidence intervals. *p*_adj_ values are one-tailed Wilcoxon signed-rank test paired by progenitor subset and then BH-corrected across the hypotheses, testing whether Epo + IL-17A results in larger progenitor populations than Epo alone. Data for this figure panel are in S3 Table. **(D)** Experimental design for evaluating IL-17A effect on hypoxia response. Triangles indicate injections of IL-17A or PBS. **(E, F)** Flow-cytometric subsets in spleen and bone marrow, respectively, at the indicated time points during hypoxia. Results are expressed as a fold-change in viable cells per gram body weight, relative to equivalent progenitor subsets in normoxia. Data pooled from four independent experiments (2 experiments each at 24 and 48 h), for a total of *n* = 6 mice for each time point for hypoxia ± IL-17A and *n* = 4 mice for normoxia. Datapoints are individual mice, lines are drawn through the means. *P*-values are Wilcoxon signed-rank test across all erythroid subsets at each time point and tissue, testing that erythroid subsets are larger in hypoxia + IL-17A than in hypoxia alone. BH correction was applied across comparisons. Data for figure panels E and F are in S4 Table.

Given the ability of IL-17A to stimulate erythropoiesis in combination with Epo, we examined whether IL-17A contributes to basal erythropoiesis. To test this, we generated mice with hematopoietic and erythroid-specific deletions of *Il17ra*. Mice with an *Il17ra*^f/f^ allele [31] were crossed with either Vav-iCre [32] or EpoR-Cre [33] mice. Quantitative-PCR (qPCR) showed poor deletion of *Il17ra* in EpoR-Cre mice (S5A Fig), however, possibly because *Il17ra* is expressed earlier than *Epor* during erythroid differentiation (Fig 1C). By contrast, *Il17ra* was deleted in over 99% of Kit^+^ and ProE cells on the Vav-iCre background, in both bone marrow and spleen (Figs 3A and S5A). There was no significant difference in hematocrit or in spleen size between IL-17ra^f/f^/ Vav-iCre mice and control Vav-iCre mice (Fig 3B), nor in any of the erythroid progenitor and precursor subsets in either bone marrow or spleen (S5B Fig). We conclude that there is no requirement for IL-17RA in the maintenance of basal erythropoiesis.

### Synergism between IL-17A and Epo is autonomous to hematopoietic cells

To determine whether the synergism *in vivo* between IL-17A and Epo is cell autonomous, we administered to IL-17ra^f/f^/ Vav-iCre and control Vav-iCre mice either Vehicle, IL-17A, Epo or Epo + IL-17A for 72 hours, following the same experimental design we previously used in wild-type mice (*n* = 6 mice per treatment/genotype combination, Fig 3C). We found no significant difference between Epo and the combined Epo + IL-17A treatment in IL-17ra^f/f^/Vav-iCre mice, while synergism was still observed in the response of both spleen and bone-marrow erythroid progenitors and precursors in control Vav-iCre mice (*p*_adj_ = 0.015, Fig 3C). We therefore conclude that the synergistic interaction between Epo and IL-17A is autonomous to hematopoietic progenitors.

### IL-17A accelerates the erythropoietic response to hypoxia

Tissue hypoxia induces an erythropoietic stress response, driven by increased Epo secretion from the kidney. The resulting higher Epo concentration in blood accelerates erythropoietic rate by promoting survival and cycling of erythroid precursors (Fig 1A). We asked whether IL-17A could stimulate erythropoiesis in the context of physiological stress, without administration of exogenous Epo.

We previously modeled erythropoietic stress by placing mice in a reduced-oxygen environment (10% O_2_, compared with 21% oxygen at sea level), finding that blood Epo levels increase and plateau at 0.03 U/ml within 24 hours of the onset of hypoxia, in turn increasing ProE and EryA in spleen and bone marrow [11]. To test whether IL-17A impacts this hypoxic stress response, we injected mice with either IL-17A (200 ng/g every 12 hours) or vehicle for 24 hours before either transferring them to a 10% O_2_ environment for up to 48 hours or allowing them to remain in normoxia. We continued IL-17A administration up until the end of the experiment (Fig 3D).

Older reports based on colony formation assays show that in response to acute erythroid stress, bone-marrow erythroid expansion is delayed relative to the spleen, possibly because bone-marrow BFU-E initially migrate to the spleen, where they contribute to rapid erythroid expansion [34–36]. Here, our flow-cytometric assessment of BFU-E and CFU-E using the “10 color stain” [5,30] (S3A Fig) is in agreement with these older studies, showing an initial decline in bone-marrow BFU-E and a slower bone-marrow erythroid expansion relative to the spleen (Fig 3E and 3F; S4 Table).

Significantly, treatment with IL-17A accelerated the erythroid response in both tissues. At 24 hours in hypoxia, IL-17A treatment increased nearly all of the spleen progenitors and precursors on the erythroid differentiation trajectory when compared with hypoxia alone (early and late CFU-E, ProE, and EryA/B/C, *p*_adj_ = 0.004, Wilcoxon signed-rank paired by cell subset, with BH correction) (Fig 3E). As an example, splenic ProE increased by 1.76 ± 0.48 fold in hypoxia, but by 5.7 ± 1.9 fold in combined hypoxia with IL-17A. In the bone marrow, the response to hypoxia became apparent at 48 hours, with IL-17A treatment further increasing erythroid progenitors and precursors (Fig 3F, *p*_adj_ = 0.004, test as above). Late CFU-E, for example, increased by 1.9 ± 0.15 fold in response to hypoxia, but by 4.1 ± 1.0 in response to hypoxia with IL-17A. Taken together, these findings show that IL-17A accelerates the erythropoietic response to hypoxia.

Of interest, we found that mice placed in hypoxia for 24 hours had a significantly increased level of IL-17A in plasma compared with normoxia (5.49 ± 2.09 pg/mL versus 1.19 ± 0.52 pg/mL, *p* = 0.03, *n* = 31 mice, S5C Fig), suggesting a potential role for IL-17A in hypoxia responses. It is unclear, however, what the hypoxia-induced IL-17A levels would be in tissue and whether this would suffice to drive erythropoiesis.

### IL-17A amplifies the transcriptional response of erythroid progenitors to Epo

**Fig 4:**
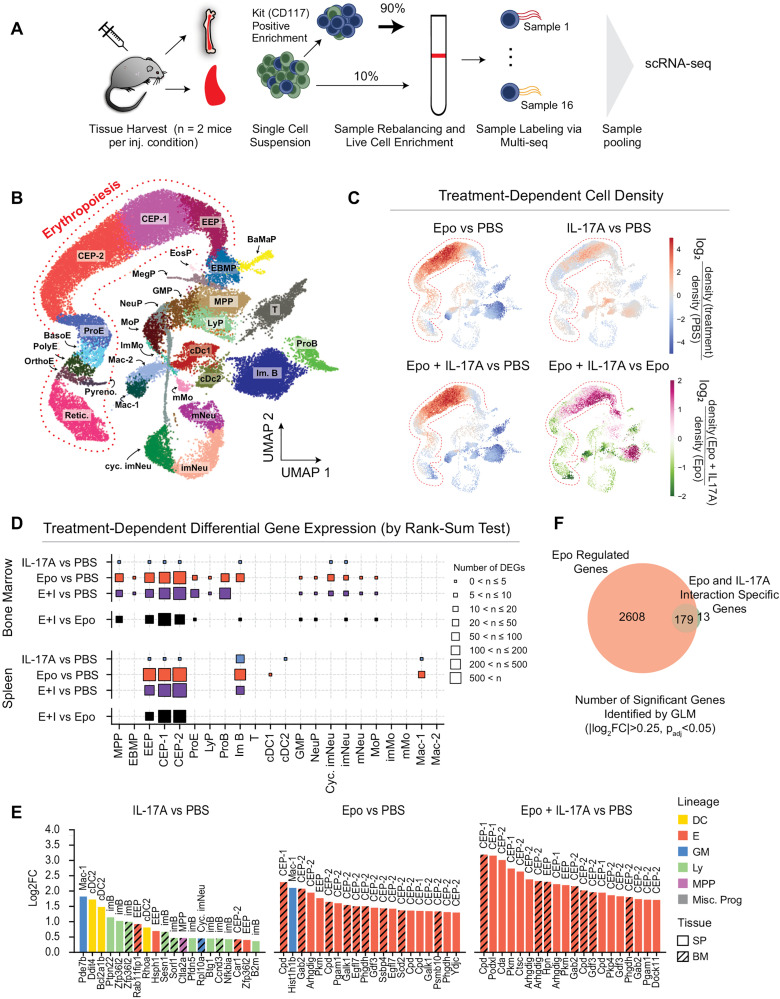
Single-cell transcriptomics of the Epo/IL-17A synergism in erythropoiesis. **(A)** Experimental workflow for scRNA-seq. Bone marrow and spleen samples (*n* = 2 mice per treatment) were enriched for Kit^+^ cells, labeled using MULTI-seq, and pooled for sequencing. **(B)** UMAP visualization of single-cell transcriptomes across conditions and tissues, colored by annotated cell states. MPP, multipotent progenitor; EBMP, erythroid-basophilic-megakaryocytic progenitor; BaMaP, basophil-mast cell progenitor; MegP, megakaryocyte progenitor; EosP, eosinophil progenitor; LyP, lymphoid progenitor; GMP, granulocyte monocyte progenitor; MoP, monocyte progenitor; imMo; immature monocyte; mMo, mature monocyte; NeuP, neutrophil progenitor; imNeu, immature neutrophil; mNeu, mature neutrophil; imB, immature B cell; proB, proB cell; T, T cell; cDC1/2, conventional dendritic cell 1/2; pDC, plasmacytoid dendritic cell; Mac, macrophage; EEP, early erythroid progenitor (BFU-e equivalent); CEP, committed erythroid progenitor (CFU-e equivalent); ProE, proerythroblast; BasoE, basophilic erythroblast; PolyE, polychromatic erythroblast; OrthoE, orthochromatic erythroblast; pyrenocytes, and reticulocytes. **(C)** Relative cell densities across the sampled RNA-Seq state space, evaluated in high dimensions and visualized on the UMAP plot. The plots are colored by the log2-transformed ratio of cell densities in the neighborhood of each sampled transcriptome, for each treatment pair as indicated. **(D)** Number of differentially expressed genes (DEGs) between cells classified to each indicated cell state across treatment conditions in bone marrow and spleen. E + I: Epo + IL-17A. DEGs are identified by a rank-sum test with BH correction, and |log2(fold-change)| > 0.25 and *p*_adj_ < 0.05. **(E)** The top 20 DEGs across conditions and cell populations by magnitude of fold-change. Bar height shows log2 fold change; colors indicate lineages: dendritic (DC), erythroid (E), granulocyte-monocyte (GM), lymphoid (Ly), multipotent progenitors (MPP), miscellaneous progenitors (Misc. Prog.). Solid bars represent spleen (SP), hashed bars represent bone marrow (BM). **(F)** Venn diagram of genes found to be significantly regulated in a generalized linear model (GLM) that accounts for separate effects of tissue-of-origin, cytokine treatment (Epo or IL-17A), and cytokine interactions (Epo and IL-17A). The overlap of Epo regulated genes and Epo-IL-17A interaction-specific genes show a large proportion of the Epo and IL-17A interaction genes are regulated by Epo alone. See S6 Fig for GLM definition and underlying results. Data for panels C–E are in S10 Table.

Our flow cytometry results showed that IL-17A does not enhance Epo’s pro-survival effect on erythroblasts (S4D Fig). What then is the mechanism underlying the synergistic action of Epo and IL-17A? To address this, we undertook scRNA-seq of bone-marrow and spleen hematopoietic cells from mice treated for 72 hours with IL-17A alone, Epo alone, combined Epo and IL-17A or vehicle, using the same experimental design as in the flow cytometry experiments above (Fig 2A). To ensure representation of rare progenitor populations, we first enriched the tissues for Kit^+^ cells, and then pooled these with 10% unenriched cells (Fig 4A). We analyzed 28,681 cells from both spleen and bone marrow (*n* = 2 mice per treatment; cell counts and markers used for cell annotation in S5 Table and S6A Fig). This data set contains a representation of the complete erythroid trajectory, from MPP to reticulocytes, as well as eight other hematopoietic lineages derived from MPPs, consistent with previous work [5,37–39] (Fig 4B).

Analysis of cell density changes along the erythroid trajectory showed that both Epo alone and the combined Epo + IL-17A treatments led to increased cell frequency of EEP (functionally BFU-E, see S3A Fig for nomenclature) and CEP-1/CEP-2 (functionally early and late CFU-E, Fig 4C), consistent with our flow-cytometry results (Fig 2). Further, differential gene expression analysis showed that most differentially expressed genes (DEGs) in response to either Epo or the combined treatment were seen in the same cell subsets (EEP and CEP, Fig 4D and S6 Table). Of note, in contrast to Epo or the combined treatments, there was little transcriptional response to IL-17A alone. Further, when compared with vehicle, the majority of DEGs in response to the combined Epo + IL17A treatment were similar to the DEGs in response to Epo alone, but their expression was consistently higher in the former, by up to 2-fold (Fig 4E). Of the top DEGs (Fig 4E and 4F), most were previously reported as Epo-responsive genes, including *Podxl*, *Cda, Cpd, Gdf3*, *Gab2*, and *Arhgdig* [5,40,41]. Of note, unlike erythroid progenitors, the immature B cells cluster shows increased cell density that is the result of stimulation with IL-17A alone and is not affected by the presence of Epo (Fig 4C).

**Fig 5:**
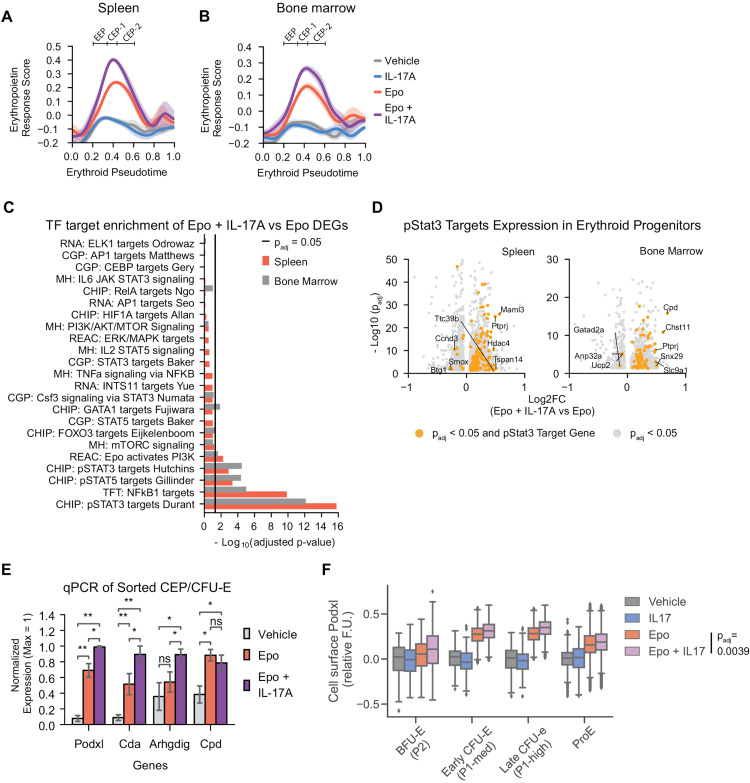
IL-17A amplifies the transcriptional response to Epo in erythroid progenitors. **(A, B)** Epo response scores for different cytokine treatments, evaluated from the scRNA-seq data (**Fig 4**) across erythroid pseudotime in spleen (A) and bone marrow (B). The pseudotime span representing erythroid progenitors (EEP and CEP) is marked. Epo response genes as in S5 Table. The observed increased expression score in response to the combined Epo + IL-17A treatment is not due to global changes in mRNA composition, as seen from expression-matched random gene set showing no change in response to the combined treatment (S6D Fig). Detailed description of Epo response score calculation can be found in Methods. **(C)** Gene set enrichment analysis of transcription factor targets from differentially expressed genes in scRNA-seq data (**Fig 4**), for cells classified as erythroid progenitors, comparing treatment with Epo + IL-17A to Epo. Line indicates a significance threshold with 5% BH-corrected p-values, calculated using Fisher’s exact test. Colors distinguish spleen (SP) and bone marrow (BM) results. **(D)** Volcano plots showing differentially expressed genes (gray points), and differentially-expressed pSTAT3 target genes (yellow points) in erythroid progenitors from spleen and bone marrow in Epo + IL-17A vs. Epo conditions. **(E)** Independent evaluation of gene expression for selected Epo-responsive gene transcripts (*Podxl, Cda, Arhgdig, Cpd*) measured by qPCR in sorted CFU-E/CEP cells (n = 4 mice per treatment, pooled from 4 independent experiments). Error bars represent the standard error. *p < 0.05, **p < 0.01, ns: not significant, one-tailed Wilcoxon rank-sum test. **(F)** Podxl cell-surface protein expression measured by flow cytometry in erythroid progenitors for each of the four treatments. Data pooled from 4 independent experiments. For early progenitors, data is mean of *n* = 3 mice per treatment; for ProE, *n* = 5 mice per treatment. Statistical significance tested with a one-tailed Wilcoxon signed-rank test across paired FACS subsets, show a statistically significant difference between treatment with Epo vs. Epo+IL-17A (*p*_adj_ = 0.0039). Data for panels A–E are in S10 Table. Data for panel F are in S11 Table.

We undertook three additional analyses of the scRNA-seq dataset, all of which similarly indicated that there was little response to IL-17A alone, and that the combined Epo + IL-17A treatment induced virtually the same set of genes as Epo alone, but to higher levels. First, we used a generalized linear model (GLM) to determine the contribution to gene expression of tissue type (spleen or bone-marrow) and of each of the cytokine treatment combinations (Figs 4F, S6B, and S6C; S7 Table). We found 192 genes that were induced as a result of the combined Epo + IL17-A treatment; significantly, of these, 179 genes were also induced in response to Epo alone; none were unique to either spleen or bone marrow. Second, we calculated an Epo-response score using genes previously shown to respond to high Epo levels [5] (S5 Table). This expression score was increased in our experiments in response to Epo alone, and further increased to even higher levels in response to the combined Epo + IL17A treatment, specifically in erythroid progenitors (EEP and CEP) in both spleen and bone marrow (Fig 5A and 5B). Third, we carried out an unbiased gene-set enrichment analysis for canonical and non-canonical Epo [42] and IL-17A [43] signaling pathway response genes (S5 Table). This suggested that STAT gene targets (both Stat3 and Stat5), which are canonical EpoR-activated pathways, are differentially enriched in response to the combined Epo + IL-17A treatment, compared with Epo alone (Fig 5C and 5D).

To validate the scRNA-seq analysis, we carried out RT-qPCR using independently-sorted CFU-E from bone marrow and spleen (4 mice per treatment), and we additionally measured cell-surface Podxl by flow cytometry. Of the four Epo-responsive genes selected for qPCR analysis (*Podxl*, *Cda*, *Arhgdig*, and *Cpd*), three were significantly elevated in CFU-E following Epo + IL-17A treatment compared with Epo alone (Fig 5E), and additionally, Podxl surface protein expression was similarly increased in BFU-E, CFU-E, and ProE (Fig 5F). Taken together, multiple lines of evidence suggest that IL-17A amplifies Epo-dependent gene expression. IL-17A does not induce an Epo-independent, unique set of genes in erythroid progenitors, whether administered alone or in combination with Epo.

### The accelerated response to hypoxia by IL-17A is associated with faster cycling

**Fig 6:**
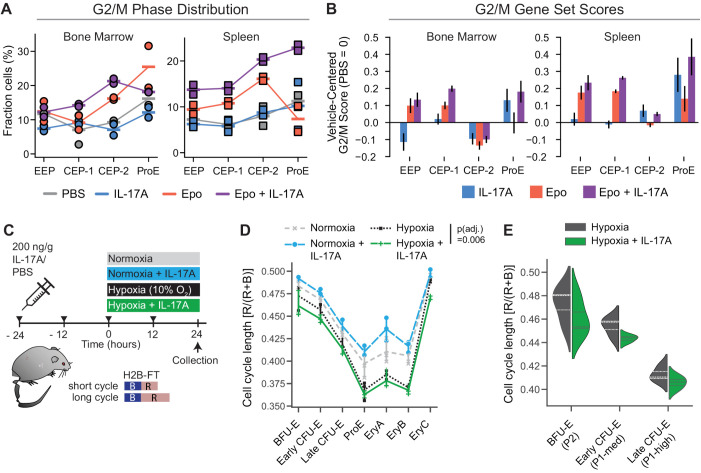
IL-17A enhances hypoxia-induced cell cycle shortening in erythroid progenitors. **(A, B)** The fraction of cells in G2/M phase (A), and expression of canonical G2/M-enriched genes (B), corresponding to each treatment. Fractions and gene set scores are inferred from scRNA-seq profiles in each of erythroid progenitor states (EEP, CEP-1, CEP-2, ProE) in bone marrow and spleen (**Fig 4**). **(C)** Experimental design for evaluating changes in erythroid progenitor cell cycle duration in response to hypoxia with or without IL-17A pre-treatment, using mice expressing the H2B-FT transgene, which undergoes blue(B)-to-red(R) maturation. The ratio of red to total (red and blue) H2B-FT fluorescence indicates cell cycle length, with a shorter cell cycle corresponding to a lower R/(R + B) ratio. **(D)** Median cell cycle length in the indicated bone marrow erythroid subsets, measured in *n* = 3 or 4 mice for each treatment. *P*_adj_-value is Wilcoxon signed-rank test (BH corrected), comparing erythroid subsets in hypoxia injected with either IL-17A or vehicle. **(E)** Distribution of cell cycle lengths in early erythroid subsets in hypoxia, injected with either IL-17A or vehicle. Data pooled from 3 mice for each treatment. An average of 1,300 cells (range: 328–5,200) per erythroid subset per treatment were analyzed. Data for panels A,B,D,E are in S10 Table.

The increase in the number of erythroid progenitors in response to the combined Epo + IL-17A treatment, as compared with Epo alone, cannot be attributed to changes in cell survival (S4D Fig). To date, there is little direct data on the cell cycle in CFU-E and BFU-E *in vivo* during the response to Epo or hypoxic stress. To investigate a potential effect on the cell cycle, we first used our scRNA-seq data to infer progenitors’ cell cycle phase following each treatment (see Methods). We found an increased number of erythroid progenitors in G2 or M following the combined treatment with Epo + IL-17A, compared with all other treatments (Fig 6A). As a complementary approach, we calculated an activity score for genes canonically expressed in G2 or M. The G2/M score increased in erythroid progenitors in response to the combined Epo + IL-17A treatment compared with Epo alone, in both bone marrow and spleen (Fig 6B).

These results indicated possible increased cycling in erythroid progenitors specifically in response to the combined Epo + IL-17A treatment compared with Epo alone, without a corresponding response to IL-17A treatment alone. To investigate this further, we examined how IL-17A impacts cycling of erythroid progenitors during hypoxic stress and in normoxia. We used mice transgenic for a live cell reporter of cell cycle duration, a chimeric histone H2B fused to a fluorescent timer protein (H2B-FT, Fig 6C) [13,44]. H2B-FT fluoresces blue when first synthesized but matures over ~1.5 hours into a red fluorescent protein. The ratio of red to total fluorescence reflects cell-cycle duration [44]. We treated H2B-FT transgenic mice with either IL-17A or PBS for 48 hours. After the initial 24 hours, half the mice from each group were transferred from normoxia to hypoxia (10% oxygen, *n* = 3 or 4 mice per group, Fig 6C–6E). Using flow cytometry, we found that IL-17A by itself appeared to slightly increase cell cycle duration in erythroid progenitors, while hypoxia resulted in a clear shortening of the cycle in all erythroid progenitor and precursor subsets. The combined treatment of IL-17A and hypoxia resulted in further cell cycle shortening, most pronounced in BFU-E and CFU-E (Fig 6D and 6E; *p*_adj_ = 0.006, Wilcoxon signed-rank test comparing all erythroid subsets in hypoxia treatment with the combined hypoxia + IL-17A treatment). We conclude that hypoxia promotes faster cycling in early (BFU-e and CFU-E) erythroid progenitors, an effect that was not previously documented; and that IL-17A further enhances this effect, thereby accelerating the erythropoietic response to hypoxia.

### A mathematical model shows advantages of IL-17A-mediated synergism through the sensitization of erythroid progenitors to Epo

Our data show that the stimulatory effect of IL-17A on erythropoiesis is entirely dependent on the presence of elevated Epo. Further, scRNA-seq analysis shows that there is no IL-17A-unique transcriptional response in erythroid progenitors, whether administered alone or in combination with Epo. Instead, IL-17A sensitizes these cells to Epo, amplifying the Epo-induced transcriptional response. A key observation is the finding that IL-17A specifically acts to amplify Epo signaling in early progenitors, as evidenced by the expression of *Il17ra* in these cells; IL-17A has little effect on the Epo transcriptional response in ETD, consistent with the lack of IL-17A/Epo synergism in ProE and Ery A/B/C survival (S4D Fig). Therefore, IL-17A alters, or ‘tunes’, the regulatory responses of early erythropoiesis to Epo, without impacting Epo regulation of ETD. What is the functional significance of this mode of synergism between IL-17A and Epo?

**Fig 7:**
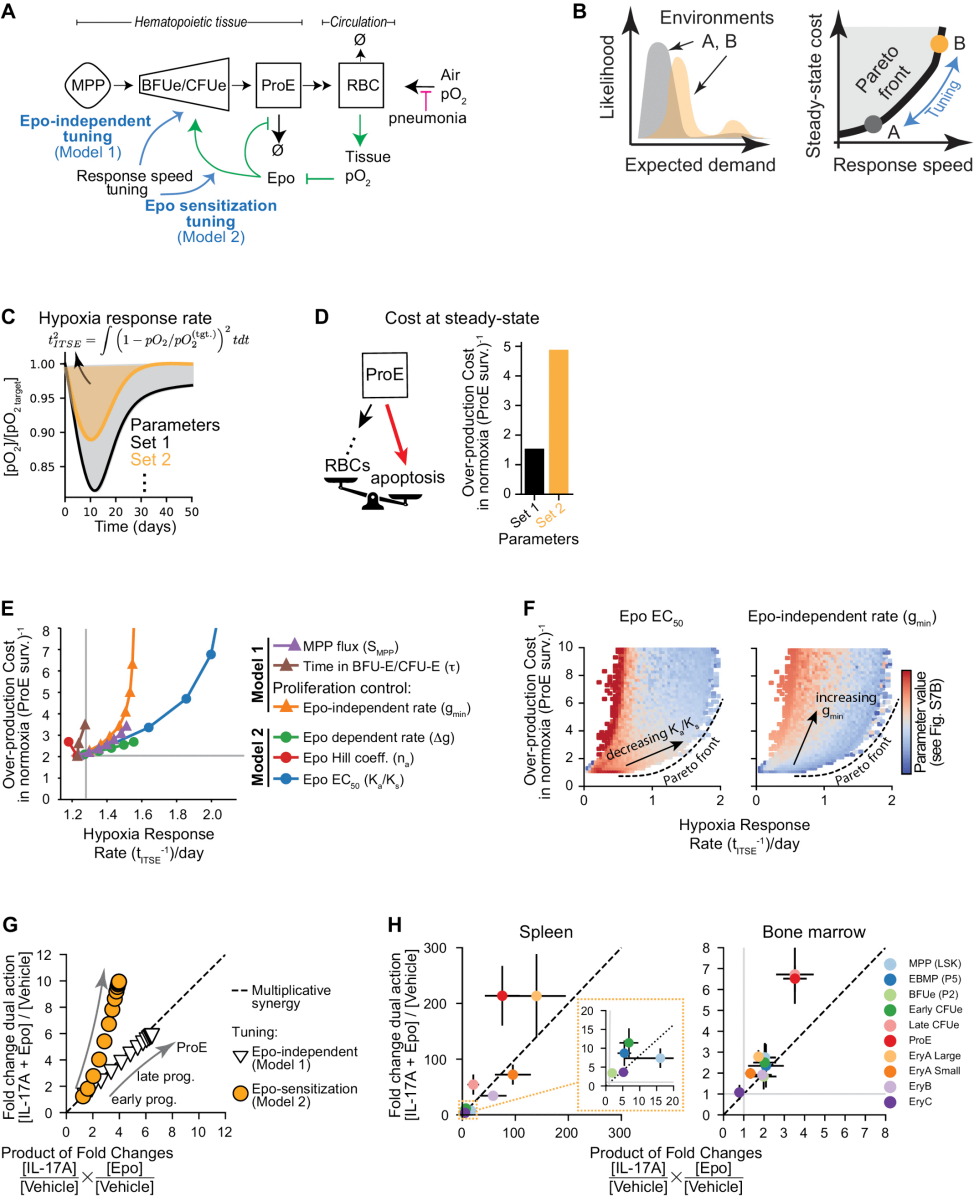
A dynamical model predicts optimal tuning mechanisms for the erythroid feedback circuit. **(A)** Schematic of a simplified dynamical systems model of erythropoiesis. Full model description is in S7 Fig and S1 Text. Mechanisms for tuning by IL-17A (blue arrows) can be summarized into two classes: Model 1 (Epo-independent tuning of progenitor number) and Model 2 (tuning Epo sensitivity of progenitors). **(B)** Illustration of performance tuning. Two environments A and B show different likelihoods of increased cell demand (left panel), and accordingly different optimal cost/reaction-speed trade-offs (right panel). Blue arrow: tuning control circuits on the Pareto front alters reaction speed at the lowest possible cost. **(C)** Examples of the dynamical model numerical solutions for pO_2_ blood concentration, following gradual hypoxic onset. Two different configurations of the system (Parameter Sets 1,2) show different recovery times (defined as the root integral time squared error ($t_{ITSE}$) of the pO_2_ levels from target). **(D)** The constitutive cost of ProE over-production during normoxia for the same parameter sets as in (C). The over-production burden results from high levels of apoptosis of ProE (shown schematically), and is defined as 1/(ProE survival). The faster response in panel C for parameter set 2 requires a higher constitutive burden. **(E)** Tuning individual control parameters from a baseline state identifies variation in their contribution to recovery speed (*t*_ITSE_)^−1^ after pO_2_ perturbation and the over-production cost [defined in (D)]. Parameters corresponding to models 1 and 2 (panel A) are denoted by triangles and circles, respectively. **(F)** Random sampling of control parameters over physiological ranges reveals a Pareto front in the response-speed/cost trade-off. The heatmaps shows the values of two parameters representative of Model 1 (Epo-independent proliferation, $g_{min}$) and Model 2 (Epo-dependent proliferation EC_50_, $K_{a}$). Color bar units and additional parameter values are given in S7E Fig. **(G)** Computational predictions for the expansion of erythroid progenitors in each of the two tuning models following Epo + IL-17A treatment as compared to multiplicative fold change of Epo and IL-17A alone. The dashed line indicates multiplicative synergy. Model 1 is implemented by modulating $g_{min}$ while model 2 is implemented by modulating $K_{a}$ (this figure) or Δg (S7F Fig). Refer to S1 Text for modeling parameters used. **(H)** Measured fold changes in cell populations from spleen and bone marrow comparing [Epo + IL-17A]/[Vehicle] treatment to the expected multiplicative fold change ([IL-17A]/[Vehicle] × [Epo]/[Vehicle]). Data points above the dashed line indicate above-multiplicative synergy. Inset shows zoomed view of lower fold changes in spleen. The same experimental data as in **Fig 2F**. Data for panels C–H are in S10 Table.

To explore this question, we developed a simplified mathematical model of erythropoiesis (Figs 7A, S7A, and S7B; S1 Text). Here, (i) MPPs give rise to transit-amplifying erythroid progenitors (BFU-E and CFU-Es); these give rise to ProE precursors that ultimately differentiate into mature RBCs; (ii) circulating RBCs carry oxygen from lung to tissue; (iii) tissue pO_2_ represses Epo production; (iv) Epo levels determine ProE survival, an established mechanism through which Epo regulates erythropoietic rate [10–12,29,45]; and (v) expansion of erythroid progenitors may be regulated through either Epo-dependent and/or Epo-independent pathways. In agreement with older work, BFU-E and CFU-E do not require Epo for survival and are relatively insensitive to Epo in the absence of stress (that is, in healthy individuals in normoxia) [2,11,46].

Through Epo-mediated negative feedback, the rate of red blood cell production—the erythropoietic rate—can be dynamically adjusted to meet changes in tissue oxygen tension. The performance characteristics of many feedback control systems can be altered, or “tuned”, so that they are optimized for specific goals, such as maximizing the speed or accuracy with which systems sense and react to their environment, minimizing resting-state fluctuations or ongoing costs in energy and resources [47–49]. In an optimized system, improving one objective may be at the expense of another (a pareto front, Fig 7B) [50,51]. In erythropoiesis, there is a clear conflict between optimizing for the system’s readiness to respond quickly to stress and maintaining low ongoing costs during steady-state basal erythropoiesis in health. Indeed, the vital importance of a fast erythropoietic response to hypoxic stress explains the costly, continuous over-production of ProE during steady state erythropoiesis, with most ProE undergoing apoptosis. Rescue of ProE from apoptosis by stress-induced Epo survival signals is a much faster mechanism of increasing ProE number than generating more of these cells through lengthy cell division and differentiation from earlier progenitors [10–12,29,45].

The above considerations suggest that the level of ProE over-production represents a balance, or compromise, between its cost during the steady-state, and the speed of response to stress. A high level of ProE over-production optimizes for a speedy response to stress, while entailing high cost during the basal steady state; conversely, low ProE over-production saves on ongoing costs but compromises the speed of response to stress (Fig 7B–7D). What then is the correct level of ProE over-production for the system? The answer may depend on the likelihood of having to mount a stress response (Fig 7B). Prior to any change in tissue oxygen tension, there may nevertheless be signals that indicate increased risk of impending hypoxic stress (a shift from environment A to environment B, Fig 7B). Under these changed circumstances, tuning the system by further optimizing for response speed may be worth the cost.

Based on our data, we propose that IL-17A is an example of a signal that anticipates increased hypoxia risk, and in turn, tunes the erythropoietic system so as to increase ProE overproduction, thereby allowing for an accelerated stress response should it be required. This suggestion is consistent with reports that IL-17A increases early in experimental pneumococcal pneumonia, and is an early marker of likely disease severity and development of acute respiratory distress syndrome in Covid-19 patients [52–54]. Further, levels of IL-17A in patients hospitalized with viral infections, including SARS-CoV2 or MERS, increase 3-–20-fold over baseline levels [54–57] and can exceed the EC_50_ for the erythroid stimulatory effect of IL-17A *in vitro* (2 ng/mL) (S8 Table).

To test how different tuning strategies might alter cost-performance trade-offs, we used numerical simulations of normoxia and of the response to a drop in oxygen tension in alveolar air (as may occur during respiratory distress). Recovery from the latter requires increased RBC production, which increases the number of circulating RBCs, thereby compensating for reduced oxygen loading of individual RBC (Fig 7C, hypoxic stress dynamics in S7C Fig). We investigated potential tuning mechanisms by using alternative sets of model parameters, and then, for each parameter set, determining the rate of response to hypoxia (quantified by the integral-time-squared error or ITSE, Fig 7C), and the rate of ProE over-production during normoxia (Fig 7D). Significantly, the model shows that ProE overproduction is essential for a fast erythropoietic response to sudden hypoxic stress (S7D Fig). It thus recapitulates the experimental observation that loss of ProE overproduction, by genetic elimination of the apoptotic mechanisms that preserve it, delays production of new RBC in response to hypoxia [12].

We proceeded to scan all model parameters by varying them one at a time (Fig 7E). This approach revealed two principal tuning mechanisms by which IL-17A might increase the number of early progenitors. First, it might do so directly and independently of Epo, by either increasing early progenitor proliferation or the rate with which they are produced from MPPs (Fig 7A, parameters controlling **Model 1**, and S7A and S7B Fig). Alternatively, IL-17A might increase the number of early progenitors by sensitizing their response to proliferative signals generated by Epo (in turn, by either reducing Epo’s EC_50_ or the maximal Epo proliferative response; Fig 7A, **Model 2** and S7A and S7B Fig).

Strikingly, analysis of the response-speed/cost trade-off for single parameters suggested, however, that Models 1 and 2 are not equivalent (Fig 7E). Varying parameters that contribute to sensitization of progenitors to Epo (**Model 2)** provided gains in response speed at a smaller associated ProE over-production cost, compared with parameters that increased progenitor number independently of Epo (**Model 1**). To evaluate whether these differences hold for all model configurations, we carried out 10^5^ additional simulations, each randomly sampling model parameter values, and evaluated the two performance goals in each case. This analysis revealed a clear pareto front in the response-speed/cost trade-off (Figs 7F and S7E). Significantly, parameters associated with Model 2 (Epo sensitization) tuned the feedback circuit along a pareto front where the speed of response to stress increased with the lowest additional burden of ProE overproduction, while the parameters associated with Model 1 (Epo-independent progenitor proliferation) did not. Thus, within our model, tuning the sensitivity of early progenitors to Epo offers the lowest possible progenitor over-production burden for a given speed of hypoxic response.

### IL-17A-mediated sensitization of progenitors to Epo results in their super-multiplicative expansion, in agreement with model predictions

In addition to differing in the response-speed/cost trade-offs, Models 1 and 2 also differ in expected changes to progenitor pool size. Model 1 (tuning through Epo-independent action) predicts that the combined Epo + IL-17A treatment would result in multiplicative synergy, with the increase in progenitor number being the product of the response to each cytokine alone (Fig 7G, on-diagonal). By contrast, Model 2 (tuning through sensitization of progenitors to Epo) predicts that expansion of erythroid progenitors in response to the combined Epo + IL-17A treatment would be “super-multiplicative”, that is, consistently larger than expected from the product of their independent actions (Figs 7G and S7F, above the diagonal). Analysis of our experimental results in Fig 2 shows that by 72 hours of the combined treatment, CFU-E, ProE, and other progenitor and erythroblast populations have expanded in a super-multiplicative way, consistent with model 2 predictions (Fig 7H).

## Discussion

Our work establishes the IL-17A cytokine as an erythropoiesis-stimulating factor that accelerates the erythropoietic stress response. Our findings also suggest broader principles that potentially explain how pleiotropic factors like IL-17A exert tissue-specific effects in response to relevant stress stimuli. Here, we find that IL-17A increases the sensitivity of erythroid progenitors to Epo, a mechanism that makes its action specific to the erythroid lineage, and improves the response speed to stress with minimal cost during the steady state.

Acting via the IL17RA/IL17RC heteromeric receptor, IL-17A synergizes with Epo in a cell-autonomous manner, stimulating proliferation of BFU-E and CFU-E erythroid progenitors. Its action results in increased production of ProE precursors above that generated by Epo alone, by 2-fold in the bone marrow and by 5-fold in the spleen. Surprisingly, we found that this striking synergism is not the result of an IL-17A-unique transcriptional response in progenitors. Indeed, when administered alone, IL-17A generates little or no transcriptional or proliferative response in erythroid progenitors. Instead, IL-17A sensitizes BFU-E and CFU-E progenitors to Epo signaling, as evidenced by its amplification of the Epo-induced transcriptional response in these cells, and by its enhancement of their proliferative response to Epo. The molecular mechanism by which IL-17A sensitizes erythroid progenitors to Epo will require further elucidation, but based on scRNA-seq transcriptional target analysis, and on experimental measurement in our previous work [5], is at least in part the result of synergistic activation of Stat3 and Stat5. Examination of DEGs suggests that sensitization is not the result of suppression of negative feedback regulators of EpoR signaling (S6 Table). Previous reports show that the sensitivity of erythroblasts to Epo can be increased by transferrin receptor ligands, either iron-loaded transferrin or polymeric IgA [58,59]. These ligands augment all three major EpoR-stimulated pathways, MAPK, PI3K, and Stat5. Our work expands these findings by showing that EpoR sensitization also plays a role in the earliest erythroid progenitors. Together, this body of work suggests that there are as yet unknown mechanisms regulating the sensitivity of EpoR signaling responses which are relevant to erythropoietic stress regulation.

We were puzzled by the lack of an IL-17A-unique, Epo-independent transcriptional response that would explain the stimulatory effect of IL-17A on erythroid progenitors. However, a dynamical model of erythropoiesis suggested a substantial advantage to the Epo-sensitization-based mode of action we uncovered for IL-17A. Thus, there is an inherent delay in the production of RBCs from HSC (~10 cell divisions or more [15,60]), that would impede a speedy response to hypoxic stress. To overcome this, the erythropoietic system over-produces ProE precursors, the majority of which undergo apoptosis in normoxia, but are also readily available should they be required in a stress response. Our dynamical model of erythropoiesis indeed confirms that continuous ProE over-production accelerates the response to stress, as does previous work that experimentally eliminated ProE over-production [12]. Therefore, erythropoiesis accommodates a trade-off between the speed of response to stress and the ongoing cost of over-producing ProE precursors. IL-17A “tunes” this trade-off by increasing the speed of response to stress, but this also inevitably increases the cost of precursor overproduction during normoxia. To understand these effects at the system level, we undertook comprehensive numerical simulations of tunable model parameters, finding that they fall into two groups: parameters that determine progenitor proliferation independently of Epo; and those that determine the sensitivity of progenitors to Epo signaling. The two mechanisms are not equivalent, however: sensitizing progenitors to Epo resulted in far lower precursor over-production cost for a given increase in response speed, compared with parameters that increased progenitor proliferation in an Epo-independent manner. IL-17A, therefore, synergizes with Epo through an efficient mechanism that minimizes costs during the steady state. This finding is also born experimentally, since we found little increase in ProE overproduction (the “cost”) in response to IL-17A alone.

What is the physiological role of IL-17A-mediated stimulation of erythropoiesis? IL-17A is an early marker in severe infections, including in pneumonia that might progress to respiratory distress [52–54]. Hypoxemia may develop progressively and is highly correlated with mortality [61–63]. IL-17A could serve to pre-emptively tune erythropoiesis so as to accelerate the speed of response to hypoxia, should it occur, reducing both the depth of incurred hypoxemia and the time to recovery. We indeed found that priming mice with IL-17A for 24 hours accelerated their erythropoietic response to subsequent hypoxia, an experimental model that may reflect the role of IL-17A during infectious pneumonia. Further work is required to determine the type and range of erythroid stress responses that are regulated by IL-17. It will be of interest to determine whether, in addition to a physiological role in the erythroid stress response, IL-17A contributes to myeloproliferative neoplasms such as polycythemia vera, where it might synergize with the constitutively active JAK2V617F in erythrocyte over-production.

In addition to IL-17A, erythroid progenitors are subject to regulation by several other pleiotropic factors, including SCF, BMP4, and glucocorticoids [18–21]. BFU-E and CFU-E progenitors, therefore, integrate multiple environmental cues that, like IL-17A, may tune the system so as to optimize its performance in a changing environment. The enhancement of EpoR signaling by IL-17A bears similarities to the functional synergism between EpoR and Kit [64,65]. Both Kit and IL-17RA are expressed by erythroid progenitors and are downregulated in ETD. Both IL-17A and SCF (Kit ligand) increase the number of Epo-dependent CFU-e colonies *in vitro* [64,66]. Both factors also increase erythropoietic rate during stress by increasing proliferation of erythroid progenitors [21,67]. However, the mechanism of SCF-mediated enhanced proliferation of erythroid progenitors is unclear. Like IL-17, SCF enhances EpoR-induced Stat5 activation [68,69]. However, there is no data at present on whether, like IL-17, SCF alters cell cycle duration. Further, unlike IL-17, SCF promotes progenitor survival [70,71]. We are not aware of studies probing the transcriptional consequences of SCF/Epo cooperative signaling in erythroid progenitors *in vivo* in the manner that we investigated here for IL-17. Most importantly, the two factors differ in that SCF/Kit signaling is essential for steady-state erythropoiesis [72,73], whereas IL-17 is dispensable, and its function is limited to accelerating erythropoietic rate in response to a subset of stress stimuli likely involved in inflammation or infection.

Although the *Il17ra* receptor is expressed broadly, we found that administration of IL-17A alone had little effect on hematopoietic cells, and that, in combination with Epo, its actions were largely specific to erythropoiesis. This specificity is explained by its mechanism of action, through sensitization of progenitors to Epo. IL-17A is documented to amplify signals of other tissue-specific factors [74–76]. Our work thus offers a template for understanding broader functions of IL-17 and potentially the functions of other pleiotropic factors. We note, however, that when amplifying inflammatory responses of granulopoiesis and lymphopoiesis, IL-17 may lead, indirectly, to secretion of inflammatory mediators such as IFN-γ and TNF-α, most of which are documented to suppress erythropoiesis [77,78]. Indeed, our finding here, that the direct actions of IL-17 on erythropoiesis are stimulatory, are especially interesting given the negative effect of most inflammatory mediators on this system [77,78]. Thus, while the specificity of action of IL-17 suggests it could be used as therapy to augment the erythropoietic stress response, IL-17’s pro-inflammatory effects might limit its erythropoiesis-stimulating applications to specific clinical settings, an area that will need to be addressed in future translational work.

## Materials and methods

### Ethics statement

All work involving mice was approved by the University of Massachusetts Chan Medical School Institutional Animal Care and Use Committee (IACUC) protocol PROTO202200017 (Socolovsky laboratory).

### *In vivo* animal studies

Mice treated with IL-17 and Epo *in vivo* were either BALB/cJ, Il17ra^f/f^/Vav-iCre (obtained by crossing Il17ra^f/f^ (B6.Cg-*Il17ra*^*tm2.1Koll*^/J) with Vav-iCre (B6.Cg-*Il17ra*^*tm2.1Koll*^/J), both obtained from the Jackson Laboratory) or Vav-iCre. Mice treated with IL-17 and hypoxia were either BALB/cJ, or mice transgenic for H2B-FT (B6;129-*Gt(ROSA)26Sor*^tm1(rtTA*M2)Jae^ *Hprt1*^tm2(tetO-mediumFT*)Sguo^/Mmjax, obtain from the Gao laboratory, Yale school of medicine). All mice were 8–12 weeks old at the time of experiments. Littermates of the same age and sex were randomly assigned to treatment groups. Both male and female mice were used in experiments.

### CFU-E colony formation assays

Adult BALB/cJ bone marrow mouse bone-marrow cells were plated at 500,000 cells/ml in methylcellulose (Iscove’s Modified Dulbecco’s Medium, 6% Bovine serum albumin (BSA), 5% fetal calf serum (FCS), 1 mg/ml human transferrin) with added Epo and IL-17 cytokines at the indicated concentrations. Colonies were counted at 72 hours after staining with diaminobenzidine to visualize hemoglobinized cells.

### Cytokine treatment in vivo

Mice were injected with cytokines subcutaneously, in a total volume of 4 microliters/g body weight. Recombinant IL-17A (made in Chinese Hamster Ovary (CHO) cells) was freshly resuspended in PBS up to 12 hours prior to use and injected at 200 ng/g body weight every 12 hours. Epo (Procrit) was injected at 0.25 IU/g every 24 hours. Vehicle- treated mice were injected with an equal volume of PBS.

### Tissue harvesting

Femur, tibiae, and spleen were harvested following euthanasia and submerged in SB5 buffer (PBS supplemented with 0.2% (w/v) bovine serum albumin (BSA), 0.08% (w/v) glucose, and 5 mM of Ethylenediaminetetraacetic acid (EDTA)). Tissue harvest methods are described in [30].

### Hypoxia model of erythropoietic stress

Mice were placed for up to 48 hours in a hypoxic environment using the BioSpherix A chamber (BioSpherix). Hypoxia was achieved by displacing oxygen with nitrogen at normal atmospheric pressure. Temperature, humidity, and carbon dioxide readings were monitored.

### Flow cytometry

All antibody panels included either Fc block or rabbit IgG (Jackson ImmunoResearch Laboratories, Item# 011-000-003). All cells were post-labeled with DAPI (Thermofisher Scientific, Item # D1306) to exclude dead cells. Reticulocytes were measured by flow-cytometric analysis of blood labeled with the Draq5 (Cell Signaling Technology, Item # 4084L) and with CD71 antibody (Biolegend, Clone RI7217). Erythroid precursors (ProE, EryA/B/C) were identified in spleen and bone marrow samples that were labeled with lineage markers (Gr1(BV786, biotin,FITC, BD Biosciences, RB6-8C5 Clone; AF700, Biolegend, RB6-8C5), Mac1 (biotin, FITC, BD Biosciences, M1/70 Clone; AF700, PE, Biolegend, M1/70 Clone), CD4 (BV510, AF700, Biolegend, RM4-5 Clone; biotin, FITC, BD Biosciences, RM4-5 Clone), CD8a (biotin, FITC, BD Biosciences, 53-6.7 Clone; AF700, Biolegend 53-6.7 Clone), CD19 (biotin, FITC, BD Biosciences, 1D3 Clone; AF700, Biolegend, 1D3/CD19 Clone; PeCy7. Invitrogen, 1D3 Clone), F4/80 (AF700, FITC, Biolegend, BM8 Clone; biotin, BD Biosciences, T45-2342 Clone), CD71 (BV421, BV510, PeCy7, Biolegend, RI217 Clone) and Ter119 (APC, biotin, BUV395, BD Biosciences, Ter119 Clone; FITC, PeCy5, Biolegend, Ter119 Clone) antibodies [4]. Early hematopoietic and erythroid progenitors (BFU-E/P2, early CFU-E/P1-medium, late CFU-E/P1-high, basophil/mast cell progenitors/P3, megakaryocytic progenitors/P4, EBMP/P5) were identified by labeling with antibodies directed at Kit (APC-Cy7, Biolegend, 2B8 Clone; AF488, Biolegend, ACK2 Clone), lineage markers, Ter119 (APC, Biotin, BUV395, BD Biosciences, Ter119 Clone; FITC, PeCy5, Biolegend, Ter119 Clone), CD71(BV421, BV510, PeCy7, Biolegend, RI217 Clone), CD55 (AF647, Biolegend, RIKO-3 Clone), CD49f (BV421, AF488, Biolegend, GoH3 clone), CD105 (AF488, PE, Biolegend, MJ7/18 Clone), CD150 (BV650, Biolegend, TC15-12F12.2 Clone), CD41(BV605, Biolegend, MWReg30 Clone) (the “10 color panel”) [5,30]. The Podxl (PE, Biolegend, 10B9 Clone) antibody was added to the 10-color stain panel in the indicated experiments. Early hematopoietic progenitors CMP, MEP, and GMP were identified using antibodies directed at Kit (APC Cy7, Biolegend, 2B8 Clone; AF488, Biolegend, ACK2 Clone), Sca1 (PerCP-Cy5.5, Biolegend, E13-161.7 Clone), lineage markers, CD34 (BV421, Biolegend, MEC14.7 Clone) CD16/32 (PeCy7, BD Biosciences, 2.4G2 Clone). Non-erythroid hematopoietic cells (‘lympho-myeloid’ subsets) were identified using antibodies directed at CD19 (FITC or biotin, BD Biosciences, 1D3 Clone; AF700, Biolegend, 1D3/CD19 Clone; PeCy7, Invitrogen, 1D3 Clone), CD4 (BV510, AF700, Biolegend, RM4-5 Clone; biotin, FITC, BD Biosciences, RM4-5 Clone), CD8a (bioitin, FITC, BD Biosciences, 53-6.7 Clone; AF700, Biolegend, 53-6.7 Clone), Mac1 (biotin, FITC, BD Biosciences, M1/70 Clone; AF700, PE, Biolegend, M1/70 Clone) and Gr1 (BV786, biotin, FITC, BD Biosciences, RB6-8C5 Clone; AF700, Biolegend, RB6-8C5). All antibody labeling was done on freshly harvested cells at 4 °C. Cells were analyzed on a Cytek Aurora cytometer with 5 Lasers. Data was analyzed using FlowJo 10.10.0.

### Flow sorting

For isolating BFU-E, CFU-E, or Kit^+^: harvested bone-marrow and spleen cells were enriched for Kit^+^ early progenitors using MojoSort Streptavidin Nanobead (Biolegend, Item #480016) bound to biotinylated anti-CD117 antibody. Enriched cells were then labeled with the “10 color panel” [5,30] antibodies and sorted on a BD FACSFusion with 5 Lasers. For isolating ProE, harvested spleen and bone-marrow were labeled with CD71 (BV421, BV510, PeCy7, Biolegend, RI217 Clone), Ter119 (APC, biotin, BUV395, BD Biosciences, Ter119 Clone; FITC, PeCy5, Biolegend, Ter119 Clone), and lineage markers and sorted or on a BD FACSFusion.

### qPCR assay for deletion efficiency of Il17ra

Genomic DNA was extracted from whole bone marrow and spleen, or from flow-sorted ProE and Kit+ cells. For each genomic DNA sample, qPCR was performed using unique primers to exons 2 or exon 3 within the ‘floxed’ regions, and to either exon 7 or exon 5 outside the “floxed” region. Deletion efficiency was calculated from ΔΔCT between the exons that are external and internal to the “floxed” region.

### IL-17A serum measurements

Murine IL-17A from blood serum was measured using the Mouse IL-17A ProQuantum Immunoassay Kit (Invitrogen Item# A46737) following the manufacturer’s protocol. The CFX96 Touch Real-Time PCR Detection System was used for the readout.

### Dynamical systems modeling of tuning in erythropoietic differentiation

Definition of the dynamical systems model shown in Figs 7 and S7 is provided in the file S1 Text, including details on generation of all modeling figure panels. Numerical simulations were performed as described in this supplement using Python (v3.10.13) with SciPy (v1.11.4), with code available at https://zenodo.org/records/17373656.

### ScRNA-seq of bone marrow and spleen cells

#### CD117-positive cell enrichment.

Harvested tissue samples were first enriched for CD117+ cells. Bone marrow and spleen cell samples were washed using Easy Sep buffer (PBS, 2% fetal bovine serum (FBS), and 1 mM EDTA) and enriched for CD117-expressing cells using EasySep Mouse CD117 Positive Selection Kit. The enrichment was performed largely following the manufacturer’s protocol; however, all room temperature steps were instead performed at 4 °C in a walk-in cold room.

#### Live cell enrichment using density centrifugation.

Following CD117 enrichment, dead cell, and debris were removed using centrifugation in Iodixanol density gradient (‘Optiprep’). Solutions of 40%, 18%, 12%, and 5% OptiPrep were prepared using weight by volume (w/v) and pre-chilled. Cells were resuspended in 0.5 mL PBS and mixed with 1 mL of 40% OptiPrep in a 5 mL FACS tube. Eighteen%, 12%, and 5% OptiPrep were sequentially and carefully overlaid on-top of the cell sample. The samples were then spun at 800*g* for 15 min with the centrifuge break off to prevent disruption of gradient. The live cell layer, which formed between the 40% and 18% interface, was collected and washed using cold PBS.

#### MULTIseq labeling of cell samples.

To reduce technical variability from sample processing, we performed MULTIseq labeling to multiplex samples for 10× Chromium-based encapsulation. Samples were labeled with MULTI-Seq Lipid-Modified Oligos (Sigma Aldritch LMO001) and unique sample barcode oligos following the MULTI-Seq protocol [5] (see S5 Table for MULTI-Seq barcodes per sample and S9 Table worksheet “Primers for Hashtags” for primer sequences used). Antibody-based hashing methods were tested for these cell samples but ultimately not used due to poor labeling of reticulocytes which lack of the hashing epitope.

#### Single-cell encapsulation and library prep.

Following MULTI-seq sample labeling, samples were pooled in equal ratios and cells were resuspended in PBS and 0.04% BSA at 1,000 cells/µL prior to cell encapsulation. To help with demultiplexing, single-injection condition pools were run individually, and an all-sample pool was run in another, for a total of five unique pools and a target total cell number of 48,000 loaded separately onto a 10× Genomics Chromium Next GEM Chip G. Samples were encapsulated using a Chromium X Instrument based on manufacturer’s instructions.

The scRNA-seq gene expression libraries were processed using the standard procedures following the Chromium Single Cell 3′ Reagents Kits User Guide (v3.1 Chemistry). The MULTI-seq barcode libraries were processed following the MULTI-seq library preparation protocol [5]. Library QC was performed using Agilent 2100 Bioanalyzer, Agilent Tape station, and KAPA Library Quantification. Libraries were sequenced on the Illumina NovaSeq 6000 using an S4 200 cycle flow cell and SP 100 cycle flow cell with a target read depth of 25,000 reads per cell for the gene expression and 5,000 reads per cell for the sample barcodes.

### scRNAseq data analysis

#### Computing environment.

Unless stated otherwise, all analyses were carried out in Python (v 3.7.12), using pandas (v1.3.5) for data manipulation, numpy (version 1.21.6) and scipy (version 1.7.3) for numerical computations, and scanpy (version 1.9.1) for handling single-cell data. These analyses were performed on Harvard Medical School’s O2 high-performance computing cluster. Computational efficiency was enhanced by parallelizing computations across cell states using the multiprocessing.

#### Data preprocessing.

The sequencing libraries were demultiplexed using Bcl2fastq (Illumina) and concatenated across multiple sequencing runs. The demultiplexed Fastq files were processed using Cellranger (v6.1.2) Gene Expression Pipeline. Alignments were performed using *M. musculus* Ensembl release 84 mm10 v1.2.0 cDNA reference. The MULTI-seq library Fastq files were processed using CITE-seq-Count tool (v1.4.5) [79] to determine sample identity of pooled cells. Sample barcode identity assignments to cells were performed using a custom python-based adaption inspired by the deMULTIplex R package [80].

Gene expression data quality control was performed using the Scanpy pre-processing pipeline with the following criteria: *sc.pp.filter_cells* was used to include cells with at least 500 unique molecular identifies (UMIs) and 200 unique genes expressed. Cells with more than 5% transcriptome contributed by Gm genes (Gm26917, Gm42418, Gm25580, Gm24139, Gm24146) or by mitochondrial genes (beginning with mt-), representing likely stressed or dead cells, were excluded from downstream analysis. Doublets were identified using Scrublet [7] and also filtered from further processing. *Sc.pp.filter_genes* was used to filter for genes that were expressed in at least 3 cells across all samples. The gene expression matrix was then globally scaled by normalizing gene expression measurements by the total UMIs per cell and multiplied by a scaling factor of 1e4 to obtain counts per ten thousand (CP10K). Gene expression values were then transformed to log(1 + CP10K).

#### Transfer learning and UMAP visualization.

For data graph embedding and visualization, we first projected the transformed data into a linear subspace learnt by carrying out PCA on an scRNA-seq dataset from a well-characterized murine hematopoiesis reference (GEO accession: GSE89754) [2]. To carry out PCA on the reference data set, we processed the raw counts following the same steps as described above and used highly variable genes (selected using scanpy’s *sc.pp.highly_variable_genes* with default parameters) to perform dimensionality reduction by PCA (for *N* = 50 components). Using the PCA loadings of the reference dataset, we then projected the new dataset collecting in this study, from all conditions, onto the reduced PC space. A kNN graph was constructed for the pooled samples using *k* = 10 neighbors and then visualized using UMAP.

#### Cell state annotations.

Cell annotations were assigned using a combination of query-based transfer learning and manual curation. Published scRNA-seq data sets containing bone marrow and splenic cells were curated and used as reference data sets [5,81,82] (GEO accessions: GSE89754, GSE132042, GSE132901, GSE261601). Each reference dataset used for cell annotation was pre-processed individually in the method described above for the reference data set used for visualization.

The new data set was projected into a PCA subspace constructed for each of these reference data sets, following the same steps as in the “*Transfer learning and UMAP visualization*” subsection above. This process resulted in four independent rounds of classification. In each case, a k-nearest neighbors (kNN) classifier (*k* = 5), using majority voting [2], within each neighborhood, was applied in each round to assign annotation labels from the corresponding reference dataset. Thus, each cell received four separate labels, one from each round of classification. After obtaining labels from multiple reference data sets, labels were confirmed by manual inspection of cell type-specific marker genes (S5 Table). In cases where annotations between the four reference sets disagreed, Leiden clustering (using *sc.tl.leiden* with resolution = 2) was performed to sub-cluster populations with ambiguous labels. Marker genes were identified for each ambiguous sub-cluster using *sc.tl.rank_gene_groups,* and a literature search was conducted to confirm the correct annotation based on marker genes.

#### Condition-dependent embedding density mapping.

To identify regions of enrichment or depletion of cell states in our data, we calculated embedding densities for each treatment (Fig 4C). A kNN graph (*k* = 200) was constructed using the PC space defined in the dimensionality reduction section. The adjacency matrix from this graph was used to estimate a probability density function for each condition.

Let $A_{i,j}=\text{n},\;\;\text{n}\in [\text{0},k-\text{1}]$ represent the number of neighbors for cell *i* that belong to treatment *j* in a neighborhood of size *k*. To control for sampling biases, we applied a two-step normalization process. We first normalized A column-wise to obtain $A_{i,j}^{*}=\frac{A_{i,j}}{\sum_{i}A_{i,j}}$. This adjusts for differences in the total number of cells per treatment, representing the neighborhood composition as a normalized probability. Next, we performed row normalization to convert the treatment-dependent neighborhood compositions into probability densities for each cell, ensuring that the contributions from all treatment within a cell’s neighborhood sum to 1. Each value, $\pi_{i,j}=\frac{A_{i,j}^{*}}{\sum_{j}A_{i,j}^{*}}$, represents the probability that a neighbor of cell i comes from treatment j.

To quantify treatment-specific enrichment relative to the control (PBS or Epo), we computed the $log_{\text{2}}$ fold change between the probability densities of the perturbed treatment and the control:


$$E_{i}=\text{Lo}g_{\text{2}}\left(\frac{\pi_{i,j=treatment}+\varepsilon }{\pi_{i,j=control}+\varepsilon }\right)$$

where $\varepsilon ={\text{1}\text{0}}^{-\text{4}}$ is a pseudocount to avoid divisions by zero.

The calculated enrichment densities per cell, $E_{i}$, were plotted on the UMAP embedding to visualize the global differences across treatments.

#### Differential gene expression analysis.

Differential gene expression analysis (Fig 4D; S6 Table) was conducted using Scanpy’s *sc.tl.rank_gene_groups* function, using a two-sided Wilcoxon rank-sum test on log-normalized gene expression values. Each biological replicate was paired with a matching control replicate. The analysis was performed on a cell state-by-cell state basis across conditions and across organs. To account for biological replicates, the raw *p*-values obtained from Wilcoxon rank-sum test were combined between replicates using Fisher’s Method. A false discovery rate (FDR) was calculated using the combined *p*-values using the BH procedure. A gene was considered differentially expressed if it had an absolute log2(FC) > 0.25 between the cytokine treatment-comparisons in both biological replicates and whose was FDR < 0.05. Mitochondrial genes, unlabeled genes (genes starting with Gm, AY, AC), and genes whose detection is high in empty droplets (indicative of ambient RNA) were excluded.

#### Generalized linear model for formal assessment of cytokine and organ contributions.

To determine the overlap of IL-17A gene expression response with Epo response while controlling for biological differences between organs (spleen versus bone marrow), we performed GLM on the scRNAseq data (S7 Table). The GLM was fit separately for each cell annotation: EEP, and two subsets of committed erythroid progenitors (CEP-1 and CEP-2). Genes expressed in fewer than 10 cells were excluded. The GLM was applied to the log(1 + CP10K) gene expression values.

A design matrix was constructed using binary indicator variables to encode metadata variables for treatment conditions and organ origin. For treatments, cells treated with IL-17 were assigned a value of 1 for the IL17 variable and 0 otherwise, similarly for EPO with the EPO variable. Organ origin was encoded with 1 representing spleen and 0 representing bone marrow (reference organ) in the Organ variable. The model has the form:


expression~1+Organ+Organ : IL17+Organ : Epo+Organ : IL17: Epo+IL17+Epo+IL17: Epo

with a gaussian noise. To identify genes whose expression is regulated by specific factors and interactions, we used a Likelihood Ratio Test comparing to nested (“null”) models defined in S6B Fig. For each nested model, we calculated *p*-values for each gene from a chi-squared test on the LRT test statistic:


$$\text{LRT}=\text{2}\times \left(\text{ln}L_{\text{full}}-\text{ln}L_{nested}\right)$$

where $\text{ln}L_{\text{full}}\text{\textbackslash{} and\textbackslash{} }\text{ln}L_{nested}$ are the log-likelihoods of the full and nested models, respectively. The *p*-values were then adjusted to control for the FDR using the BH procedure. Correction was performed separately for each cell state. All statistical analyses were conducted using Python (version 3.9.19), using pandas (version 1.4.3) for data manipulation, numpy (version 1.23.0) and scipy (version 1.11.0) for numerical computations, and scanpy (version 1.9.8) for handling single-cell data. The statsmodels package (version 0.14.3) was used for fitting GLMs, using sub-routine *GLM.*

#### Pseudotime analysis.

Erythroid pseudotime analysis (Fig 5A and 5B) was performed on a subset of the data that only includes cells annotated as: MPP, EBMP, EEP, CEP-1, CEP-2, ProE, BasoE, PolyE, OrthoE, and Retic. Pseudotime was assigned to each cell using Scanpy’s *scanpy.tl.dpt*, with a randomly-selected MPP was used as the root cell to define the start of the trajectory.

#### Epo response score and cell cycle phase response score.

Gene sets were defined from previously published work and are given in S5 Table (worksheets “Gene Sets Used in Paper” and “Gene Set Gene List”). The Epo response gene set (Fig 5A and 5B) was obtained from DEGs from Epo-stimulated progenitors in Tusi and colleagues [2], and the cell cycle G2/M phase gene set (Fig 6A and 6B) was selected from Whitfield and colleagues [83]. For each of these two gene sets, scores (Figs 5A, 5B, 6A, and 6B) were calculated using the *scanpy.tl.score_genes* function, providing gene expression after pre-processing to log(1 + CP10K) (see Data preprocessing above), and the following parameter values: *ctrl_size*=*N* where *N is* is equal to the size of the test gene set; *gene_pool*=*all_non_set_genes,* where *all_non_set_genes* is the set of all genes excluding the test gene set.

To visualize treatment effects (e.g., IL-17A, Epo, or Epo + IL-17A), scores were centered by subtracting the vehicle (PBS) group mean within each cell state and organ. Because the data were log-transformed, subtraction was performed directly. Standard error was propagated using the formula for independent errors:


$$\text{SEdiff}=\sqrt{{\left(\text{SEtreatment}\right)}^{\text{2}}+{\left({\text{SE}}_{\text{PBS}}\right)}^{\text{2}}}$$

#### Gene set enrichment analysis of transcription factor targets.

Gene set enrichment analysis (Fig 5C and 5D) was carried out between cells from Epo + IL-17A-treated mice and Epo-treated mice. Only cells annotated as erythroid progenitors (EEP, CEP-1, CEP-2) were included in analysis. We used transcription factor target gene sets collected from literature search of CHIP-seq and RNA-seq publications, as well as MSigDB v5.1 curated sets (Hallmark and C2 chemical and genetic perturbations, C3 transcription factor targets) [84] (see S5 Table for gene sets, worksheets “Gene Sets Used in Paper” and “Gene Set Gene List”).

DEGs were identified as described in section Differential gene expression analysis above. Prior to set-enrichment analysis, ribosomal genes, mitochondrial genes, and predicted genes (genes starting with “Gm”) were excluded from the input. Set enrichment was determined using Fisher’s exact test (one-tailed) and the resulting *p*-values were then adjusted using the BH procedure across all gene sets to control for FDR.

#### Cell cycle phase assignment.

For Fig 6A, we further performed cell cycle phase assignments adapting methods described in Tirosh and colleagues [85]. Briefly, a score was calculated for each cell cycle phase (G1/S, S, G2, G2/M, M/G1) scores as described above, with gene sets given in S5 Table (worksheets “Gene Sets Used in Paper” and “Gene Set Gene List”). Each cell cycle phase score was separately mean-centered and standardized across all cells. Cells with phase scores across all phases below 0 were assigned to G0 as they cannot be clearly assigned to a cell cycle phase from this analysis. We assign the remaining cells to the phase which the phase score is the highest across all the scores.

### RNA isolation and RT-qPCR of sorted CFU-Es (gate P1)

Due to the high level of ambient RNA present in FBS, sorted cells were washed twice in large volumes (4.5 mL) of ice-cold staining buffer to minimize RNA contamination. After washing, cells were pelleted by centrifugation, and the supernatant was removed. Cell pellets were flash-frozen and stored at −80 °C until further analysis. Total RNA was extracted from frozen pellets using the RNeasy Micro Kit according to the manufacturer’s instructions. Whole-transcriptome pre-amplification was carried out prior to qPCR as follows: cDNA synthesis was performed using Maxima H Minus Reverse Transcriptase in the presence of a poly-T oligo and template-switching oligo. Whole-transcriptome cDNA amplification was performed using KAPA HiFi HotStart Ready Mix and cDNA amplification primers following the manufacturer’s protocol. Subsequently, qPCR was carried out on the amplified cDNA using KAPA SYBR Fast at a final reaction volume of 10 µL, containing 2 µL of cDNA template, 5 µL of SYBR Green master mix, and 200 nM each of forward and reverse primers for the genes of interest. The primer sequences for each gene as well as the sample prep primers are listed in S9 Table, worksheet “qPCR Primers for Epo Response”. Measurements were performed in either 96-well or 384-well qPCR plates. For 96-well plates, reactions were run on a CFX96 Touch Real-Time PCR Detection System. For 384-well plates, reactions were run on a QuantStudio 7 Flex Real-Time PCR System. All reactions were performed in technical triplicates.

## Quantification and statistical analysis

### Statistical tests and statistical significance

Statistical tests are indicated in the figure, and text of each experiment, and S2, S4, S6, and S7 Tables. All tests that are part of multiple hypotheses were corrected for false discovery using the BH procedure or Benjamini–Yekoutieli approach as stated in text, with adjusted *p*-values given as *p*_adj_. Tests considered significant at a 5% FDR (*p*_adj_ < 0.05), or *p* < 0.05 for isolated tests.

## Supporting information

S1 Fig IL-17 cytokine activity in the CFU-e assay decays with the age of the IL17 lyophilized preparation. CFU-E assays were carried out as in Fig 1B Fig 1E. Lyophilized protein was purchased from R&D Systems. The time interval between bottling of the lyophilized protein by the manufacturer, and its resuspension prior to use, varied for different protein lots. Activity is lost if the lyophilized protein was bottled >365 days prior to the day of resuspension. In addition, once the protein is resuspended, its activity declined rapidly after 30 days. All IL-17 protein ligands (both lyophilized and resuspended) were stored at −80 °C. Data for this figure are in S1 Table.
(TIF)

S2 Fig Flow cytometric analysis of erythroid precursors and lympho-myeloid cells in mice treated with Epo and IL-17A. **(A)** Pharmacokinetics of IL-17A *in vivo*. 2 mice per dose were injected with the indicated dose of IL-17A (50 and 200 ng/g). Blood was obtained from tail bleeds, and IL-17A was measured in plasma in small volumes of plasma using immunoPCR. The figure is reproduced with permission from [27]. **(B–D)** Data underlying Fig 2F, broken out by mouse: (B) Flow-cytometric analysis of erythroid precursors in terminal differentiation. Freshly harvested bone marrow and spleen cells were labeled with CD71 and Ter119 antibodies and immediately analyzed to identify ProE, EryA/B/C subsets. FACS gates as in [29]. Data points correspond to individual mice, and box plots are defined as in Fig 2B. (C, D) Flow-cytometric analysis of lymphoid and myeloid subsets in bone marrow and spleen. **(E, F)** Loadings of each of the FACS subsets for the first two principal components of the PCA analysis in Fig 2G and 2H. The same graph as in Fig 2H, indicating tissue origin of each FACS subset (E) and the full identity of each (F). Data for panels A–F are in S2 Table.
(TIF)

S3 Fig Flow cytometric analysis of early hematopoietic and erythroid progenitors in mice treated with Epo and IL-17A. **(A)** FACS gating strategy for identifying early hematopoietic and erythroid progenitors [5,30] (the “10 color stain”). Corresponding FACS gates, functionally defined progenitors, and single-cell RNA-seq (scRNA-seq) clusters are shown on the right. Correspondence between the three modalities of defining each progenitor type was previously shown in Tusi and colleagues [5]. **(B–E)** Data underlying Fig 2F, broken out by mouse. (B–D) Summary data of bone marrow (BM) and spleen cells labeled with the ‘10 color stain’ antibody panel. Data points correspond to individual mice and box plots are defined as in Fig 2B. (E) Summary data of BM and spleen cells labeled with CD34, CD16/32 antibodies. CMP, common myeloid progenitor; MEP, megakaryocytic/erythrocytic progenitor; GMP, granulocytic/monocytic progenitor. Data for panels A–E are in S2 Table.
(TIF)

S4 Fig Complete blood count (CBC) and erythroblast survival measurements in mice treated with Epo and IL-17A. **(A–C)** CBC results from experiments described in Fig 2. Blood was obtained by cardiac puncture immediately following culling. Data points correspond to individual mice and box plots are defined as in Fig 2B. **(D)** Flow-cytometric analysis of apoptosis in erythroid precursors *in vivo*. Annexin V labeling of cells in each of the erythroid subsets shown in S2B Fig. Data for panels A–D are in S2 Table.
(TIF)

S5 Fig Conditional deletions of *Il17ra* and measurement of IL-17A in plasma during hypoxia. **(A)** qPCR analysis of *Il17ra* deletion assessed using whole bone marrow or whole spleen. Two alternative primer sets gave similar results: either primers to exons 3 and 5, or exons 2 and 7. For Vav-iCre-mediated deletion, deletion efficiency in whole tissue was less efficient than in sorted hematopoietic cells (either Kit^+^ or ProE), see Fig 3. EpoR-Cre-mediated deletion was poor. **(B)** Erythroid progenitors and precursors in Vav-iCre and Il17ra^f/f^/ Vav-iCre, analyzed by flow cytometry using CD71/Ter119 and the “10 color stain” panels (see S3 Fig). **(C)** Measured blood serum concentrations of IL-17A in mice in normoxia and following 24 hours of exposure to hypoxia following the treatment schema in Fig 3D. Measurements are carried out by immunoPCR along with recombinant murine IL-17A as a standard curve. Data points correspond to individual mice, and box plots are defined as in Fig 2B. The increase in IL-17A in hypoxia is statistically significant (Wilcoxon rank-sum test, one-tailed *p*-value <0.05) but still well below the EC_50_ of erythroid progenitors seen in CFU-E assays. Data for all panels are in S12 Table.
(TIF)

S6 Fig Annotation of cell states in the scRNA-seq data and analysis of their transcriptional response. **(A)** Heatmap showing expression of cell type-specific marker genes (columns) across cell clusters annotated to different hematopoietic cell states (rows). The genes were selected from literature as markers of cell states, as shown by their group label at the top. Marker gene list and the corresponding origins can be found in S5 Table. The diagonal pattern demonstrates marker gene specificity for their respective cell populations. Expression values are the mean of z-score standardized values. **(B)** Definition of the generalized linear model (GLM) and the nested null models used to dissect gene expression dependency on cytokine treatments and tissue/organ-of-origin for each gene, supporting Fig 4F. The full model incorporates all possible interactions between organ/tissue (spleen versus bone marrow), Epo, and IL-17A treatments. The nested models remove terms to enable statistical significance testing for different terms (Likelihood Ratio Tests followed by multiple hypothesis correction across all genes, see Methods). The biological interpretation and corresponding null model formula are shown for each comparison. **(C)** Distribution of significantly regulated genes (*p*_adj_ < 0.01, |log_2_FC| > 0.25) from applying the GLM to CEP-1. The six bars correspond to testing against each of the null models tabulated in (B). Black bars indicate genes uniquely regulated in a single comparison, while blue bars show genes shared across multiple comparisons. The analysis reveals that Epo treatment and tissue-specific differences are the dominant sources of transcriptional variation. The IL-17A and Epo interaction showed limited unique gene regulation (10 unique genes), with most regulated genes shared with other comparisons. Similar distributions were observed across other erythroid populations. The number of genes which are overlapping between Epo treatment and IL-17A and Epo interaction are shown in Fig 4F.**D** Top: comparison of changes in the expression of Epo response genes across erythroid progenitor states. Red dots indicate genes with significant differential expression (*p*_adj _< 0.05) in either Epo + IL-17A vs. PBS or Epo vs. PBS comparisons. These Epo response genes (S5 Table, from Tusi and colleagues [5]) were used to calculate response scores in Fig 5A. Select genes are labeled. Dotted lines indicate *y* = *x*. Log2(FC) = log2(fold-change). Bottom: the same analysis repeated for randomly-selected expression-matched control genes shows that the increase seen in Epo response is specific. The data for panels A, C, D are in S12 Table.
(TIF)

S7 FigModeling the response-speed/cost trade-off in erythropoietic feedback control.**(A)** Diagram of the dynamical systems model of erythropoietic feedback control. Black arrows indicate cell flux. Green arrows indicate regulatory links. Symbols in green show parameters associated with different model components. The full mathematical model is defined in S1 Text. **(B)** Table defining the model parameters in (A). **(C)** The dynamics of lung absorbance used to model in disease-associated hypoxic onset in Fig 7 and all subsequent analyses (Fig 7D–7F, and panels 7D, 7E below). The graphs show the value of model parameter for oxygen absorbance through the lung, α(t), as defined in S1 Text (Eq. [10]). **(D)** The model recapitulates a requirement for ProE apoptosis in accelerating hypoxic response rates, defined as in Fig 7C. The orange star denotes the response rate when $K_{a}/K_{s}\gg \text{1}$, $g_{min}=\text{0}.\text{3}/\text{day},\Delta g=\text{0}.\text{9}/\text{day}$, corresponding to a case where all ProE survive and Epo exclusively regulates cell proliferation. The blue curve corresponds to the same Epo regulation of progenitors, but now with $K_{a}/K_{s}=\text{2}.\text{0}$ corresponding to Epo regulating both response rate and survival. Variation along the blue curve represents varying values of the Epo-independent proliferation rate $g_{min}$, with higher values leading to high apoptosis rates. **(E)** Random sampling of model parameters in ${\text{1}\text{0}}^{\text{5}}$ simulations reveal different trade-offs in overproduction cost and response rate across parameters. This panel extends Fig 7F. Parameters were sampled over the intervals: $g_{min}\in [\text{0}.\text{3},\text{1}.\text{3}]/\text{day}$, Δg∈[0.3,1.3]/day, ${\text{K}}_{a}/{\text{K}}_{s}\in [\text{0}.\text{0}\text{5},\text{2}\text{0}]$, $n_{a}\in [\text{1},\text{3}]$. **(F)** Computational predictions for the expansion of erythroid progenitors in each of the two Models following Epo + IL-17A treatment as compared to multiplicative fold change of Epo and IL-17A alone. Pareto optimal parameters were used to define baseline. The action of model 2 is defined here as modulation of Δg. The dashed line indicates multiplicative synergy. Refer to S1 Text for modeling parameters used. The data for panels C–F are in S12 Table.(TIF)

S1 Text Dynamical systems modeling. Definition of the dynamical systems model shown in Figs 7 and S7, including details on generation of all modeling figure panels.
(DOCX)

S1 Table Data for Figs 1B and S1. (XLSX)

S2 Table Data for Figs 2B, 2C, S2A–S2F, S3A–S3E, and S4A–S4D.
(XLSX)

S3 Table Data for Fig 3C. (XLSX)

S4 Table Data for Fig 3E. (XLSX)

S5 Table scRNA-sequencing analysis information, associated with Figs 4 and 5. Includes library indices, MultiSeq Barcode indices, Marker genes, gene sets, and associated sources. (XLSX)

S6 Table Differentially expressed genes, associated with Figs 4 and 5. (XLSX)

S7 Table Generalized linear model of gene expression, associated with Figs 4, S6B, and S6C. (XLSX)

S8 Table Literature citations of IL-17A measurements in humans. (DOCX)

S9 Table **Primer sequences.** Primers for used for MULTI-seq, for RT-qPCR of Epo response genes and for testing conditional deletion of IL-17RA.
(XLSX)

S10 Table Data for Figs 1C, 3a, 3b, 4C–4E, 5A–5E, 6A, 6B, 6D, 6E, and 7E–7H.
(XLSX)

S11 Table Data for Fig 5F. (XLSX)

S12 Table Data for S5A–S5C, S6A, S6C, S6D, and S7C–S7F Figs. (XLSX)

## Abbreviations

BFU-E: burst-forming erythroid units
BSA: bovine serum albumin
CFU-E: colony-forming erythroid units
DEGs: differentially expressed genes
EBMPs: erythroid/megakaryocytic/basophil-mast cell progenitors
EDTA: Ethylenediaminetetraacetic acid
Epo: erythropoietin
EpoR: Epo receptor
ETD: erythroid terminal differentiation
FBS: fetal bovine serum
FDR: false discovery rate
GLM: generalized linear modelling
IL17RA: IL-17 receptor A chain
kNN: k-nearest neighbors
MPPs: multipotent progenitors
PBS: phosphate-buffered saline
PC: principal component
PCA: principal component analysis
ProE: proerythroblasts
qPCR: quantitative-PCR
SCF: stem-cell factor
scRNA-seq: single-cell RNA sequencing
UMIs: unique molecular identifies

## References

[1] Sender R, Fuchs S, Milo R. Revised estimates for the number of human and bacteria cells in the body. PLoS Biol. 2016;14(8):e1002533. doi: 10.1371/journal.pbio.1002533 27541692 PMC4991899

[2] Koulnis M, Porpiglia E, Hidalgo D, Socolovsky M. Erythropoiesis: from molecular pathways to system properties. Adv Exp Med Biol. 2014;844:37–58. doi: 10.1007/978-1-4939-2095-2_3 25480636

[3] Erslev A. Humoral regulation of red cell production. Blood. 1953;8(4):349–57. doi: 10.1182/blood.v8.4.349.349 13032205

[4] Erslev AJ. Clinical erythrokinetics: a critical review. Blood Rev. 1997;11(3):160–7. doi: 10.1016/s0268-960x(97)90011-4 9370047

[5] Tusi BK, Wolock SL, Weinreb C, Hwang Y, Hidalgo D, Zilionis R, et al. Population snapshots predict early haematopoietic and erythroid hierarchies. Nature. 2018;555(7694):54–60. doi: 10.1038/nature25741 29466336 PMC5899604

[6] Palis J. Primitive and definitive erythropoiesis in mammals. Front Physiol. 2014;5:3. doi: 10.3389/fphys.2014.00003 24478716 PMC3904103

[7] Pop R, Shearstone JR, Shen Q, Liu Y, Hallstrom K, Koulnis M, et al. A key commitment step in erythropoiesis is synchronized with the cell cycle clock through mutual inhibition between PU.1 and S-phase progression. PLoS Biol. 2010;8(9):e1000484. doi: 10.1371/journal.pbio.1000484 20877475 PMC2943437

[8] Jacobson LO, Goldwasser E, Fried W, Plzak L. Role of the kidney in erythropoiesis. Nature. 1957;179(4560):633–4. doi: 10.1038/179633a0 13418752

[9] Takeda K, Aguila HL, Parikh NS, Li X, Lamothe K, Duan L-J, et al. Regulation of adult erythropoiesis by prolyl hydroxylase domain proteins. Blood. 2008;111(6):3229–35. doi: 10.1182/blood-2007-09-114561 18056838 PMC2265459

[10] Koury MJ, Bondurant MC. Erythropoietin retards DNA breakdown and prevents programmed death in erythroid progenitor cells. Science. 1990;248(4953):378–81. doi: 10.1126/science.2326648 2326648

[11] Koulnis M, Porpiglia E, Porpiglia PA, Liu Y, Hallstrom K, Hidalgo D, et al. Contrasting dynamic responses in vivo of the Bcl-xL and Bim erythropoietic survival pathways. Blood. 2012;119(5):1228–39. doi: 10.1182/blood-2011-07-365346 22086418 PMC3277355

[12] Koulnis M, Liu Y, Hallstrom K, Socolovsky M. Negative autoregulation by Fas stabilizes adult erythropoiesis and accelerates its stress response. PLoS One. 2011;6(7):e21192. doi: 10.1371/journal.pone.0021192 21760888 PMC3132744

[13] Hidalgo D, Bejder J, Pop R, Gellatly K, Hwang Y, Maxwell Scalf S, et al. EpoR stimulates rapid cycling and larger red cells during mouse and human erythropoiesis. Nat Commun. 2021;12(1):7334. doi: 10.1038/s41467-021-27562-4 34921133 PMC8683474

[14] Wu H, Liu X, Jaenisch R, Lodish HF. Generation of committed erythroid BFU-E and CFU-E progenitors does not require erythropoietin or the erythropoietin receptor. Cell. 1995;83(1):59–67. doi: 10.1016/0092-8674(95)90234-1 7553874

[15] Gregory CJ. Erythropoietin sensitivity as a differentiation marker in the hemopoietic system: studies of three erythropoietic colony responses in culture. J Cell Physiol. 1976;89(2):289–301. doi: 10.1002/jcp.1040890212 987043

[16] Peschle C, Migliaccio AR, Migliaccio G, Ciccariello R, Lettieri F, Quattrin S, et al. Identification and characterization of three classes of erythroid progenitors in human fetal liver. Blood. 1981;58(3):565–72. doi: 10.1182/blood.v58.3.565.bloodjournal583565 7259837

[17] Porpiglia E, Hidalgo D, Koulnis M, Tzafriri AR, Socolovsky M. Stat5 signaling specifies basal versus stress erythropoietic responses through distinct binary and graded dynamic modalities. PLoS Biol. 2012;10(8):e1001383. doi: 10.1371/journal.pbio.1001383 22969412 PMC3433736

[18] Bauer A, Tronche F, Wessely O, Kellendonk C, Reichardt HM, Steinlein P, et al. The glucocorticoid receptor is required for stress erythropoiesis. Genes Dev. 1999;13(22):2996–3002. doi: 10.1101/gad.13.22.2996 10580006 PMC317156

[19] Broudy VC. Stem cell factor and hematopoiesis. Blood. 1997;90(4):1345–64. doi: 10.1182/blood.v90.4.1345 9269751

[20] Lenox LE, Perry JM, Paulson RF. BMP4 and Madh5 regulate the erythroid response to acute anemia. Blood. 2005;105(7):2741–8. doi: 10.1182/blood-2004-02-0703 15591122

[21] Perry JM, Harandi OF, Paulson RF. BMP4, SCF, and hypoxia cooperatively regulate the expansion of murine stress erythroid progenitors. Blood. 2007;109(10):4494–502. doi: 10.1182/blood-2006-04-016154 17284534 PMC1885504

[22] Cua DJ, Tato CM. Innate IL-17-producing cells: the sentinels of the immune system. Nat Rev Immunol. 2010;10(7):479–89. doi: 10.1038/nri2800 20559326

[23] Gaffen SL. Structure and signalling in the IL-17 receptor family. Nat Rev Immunol. 2009;9(8):556–67. doi: 10.1038/nri2586 19575028 PMC2821718

[24] Li X, Bechara R, Zhao J, McGeachy MJ, Gaffen SL. IL-17 receptor-based signaling and implications for disease. Nat Immunol. 2019;20(12):1594–602. doi: 10.1038/s41590-019-0514-y 31745337 PMC6943935

[25] Veldhoen M. Interleukin 17 is a chief orchestrator of immunity. Nat Immunol. 2017;18(6):612–21. doi: 10.1038/ni.3742 28518156

[26] Wright JF, Bennett F, Li B, Brooks J, Luxenberg DP, Whitters MJ, et al. The human IL-17F/IL-17A heterodimeric cytokine signals through the IL-17RA/IL-17RC receptor complex. J Immunol. 2008;181(4):2799–805. doi: 10.4049/jimmunol.181.4.2799 18684971

[27] Wu QC, Lee J, Swaminathan A, Winward A, Hwang Y, Socolovsky M, et al. Linker minimization and characterization of Fc-fused interleukin-17A for increased in vivo half-life. Protein Eng Des Sel. 2025;38:gzaf009. doi: 10.1093/protein/gzaf009 40626948 PMC13396947

[28] Socolovsky M, Nam H, Fleming MD, Haase VH, Brugnara C, Lodish HF. Ineffective erythropoiesis in Stat5a(-/-)5b(-/-) mice due to decreased survival of early erythroblasts. Blood. 2001;98(12):3261–73. 11719363 10.1182/blood.v98.12.3261

[29] Liu Y, Pop R, Sadegh C, Brugnara C, Haase VH, Socolovsky M. Suppression of Fas-FasL coexpression by erythropoietin mediates erythroblast expansion during the erythropoietic stress response in vivo. Blood. 2006;108(1):123–33. doi: 10.1182/blood-2005-11-4458 16527892 PMC1895827

[30] Swaminathan A, Hwang Y, Winward A, Socolovsky M. Identification and isolation of burst-forming unit and colony-forming unit erythroid progenitors from mouse tissue by flow cytometry. J Vis Exp. 2022;(189):10.3791/64373. doi: 10.3791/64373 36408979 PMC12879316

[31] Kumar P, Monin L, Castillo P, Elsegeiny W, Horne W, Eddens T, et al. Intestinal Interleukin-17 receptor signaling mediates reciprocal control of the gut microbiota and autoimmune inflammation. Immunity. 2016;44(3):659–71. doi: 10.1016/j.immuni.2016.02.007 26982366 PMC4794750

[32] de Boer J, Williams A, Skavdis G, Harker N, Coles M, Tolaini M, et al. Transgenic mice with hematopoietic and lymphoid specific expression of Cre. Eur J Immunol. 2003;33(2):314–25. doi: 10.1002/immu.200310005 12548562

[33] Heinrich AC, Pelanda R, Klingmüller U. A mouse model for visualization and conditional mutations in the erythroid lineage. Blood. 2004;104(3):659–66. doi: 10.1182/blood-2003-05-1442 15090451

[34] Kato M, Kato Y, Sugiyama Y. Mechanism of the upregulation of erythropoietin-induced uptake clearance by the spleen. Am J Physiol. 1999;276(5):E887-95. doi: 10.1152/ajpendo.1999.276.5.E887 10329983

[35] Hara H, Ogawa M. Erthropoietic precursors in mice with phenylhydrazine-induced anemia. Am J Hematol. 1976;1(4):453–8. doi: 10.1002/ajh.2830010410 1008057

[36] Goris H, Bungart B, Loeffler M, Schmitz S, Nijhof W. Migration of stem cells and progenitors between marrow and spleen following thiamphenicol treatment of mice. Exp Hematol. 1990;18(5):400–7. 2338129

[37] Pellin D, Loperfido M, Baricordi C, Wolock SL, Montepeloso A, Weinberg OK, et al. A comprehensive single cell transcriptional landscape of human hematopoietic progenitors. Nat Commun. 2019;10(1):2395. doi: 10.1038/s41467-019-10291-0 31160568 PMC6546699

[38] Dahlin JS, Hamey FK, Pijuan-Sala B, Shepherd M, Lau WWY, Nestorowa S, et al. A single-cell hematopoietic landscape resolves 8 lineage trajectories and defects in Kit mutant mice. Blood. 2018;131(21):e1–11. doi: 10.1182/blood-2017-12-821413 29588278 PMC5969381

[39] Velten L, Haas SF, Raffel S, Blaszkiewicz S, Islam S, Hennig BP, et al. Human haematopoietic stem cell lineage commitment is a continuous process. Nat Cell Biol. 2017;19(4):271–81. doi: 10.1038/ncb3493 28319093 PMC5496982

[40] Sathyanarayana P, Menon MP, Bogacheva O, Bogachev O, Niss K, Kapelle WS, et al. Erythropoietin modulation of podocalyxin and a proposed erythroblast niche. Blood. 2007;110(2):509–18. doi: 10.1182/blood-2006-11-056465 17403918 PMC1924484

[41] Gillinder KR, Tuckey H, Bell CC, Magor GW, Huang S, Ilsley MD, et al. Direct targets of pSTAT5 signalling in erythropoiesis. PLoS One. 2017;12(7):e0180922. doi: 10.1371/journal.pone.0180922 28732065 PMC5521770

[42] Kuhrt D, Wojchowski DM. Emerging EPO and EPO receptor regulators and signal transducers. Blood. 2015;125(23):3536–41. doi: 10.1182/blood-2014-11-575357 25887776 PMC4458796

[43] Monin L, Gaffen SL. Interleukin 17 family cytokines: signaling mechanisms, biological activities, and therapeutic implications. Cold Spring Harb Perspect Biol. 2018;10(4):a028522. doi: 10.1101/cshperspect.a028522 28620097 PMC5732092

[44] Eastman AE, Chen X, Hu X, Hartman AA, Pearlman Morales AM, Yang C, et al. Resolving cell cycle speed in one snapshot with a live-cell fluorescent reporter. Cell Rep. 2020;31(12):107804. doi: 10.1016/j.celrep.2020.107804 32579930 PMC7418154

[45] Kelley LL, Koury MJ, Bondurant MC, Koury ST, Sawyer ST, Wickrema A. Survival or death of individual proerythroblasts results from differing erythropoietin sensitivities: a mechanism for controlled rates of erythrocyte production. Blood. 1993;82(8):2340–52. doi: 10.1182/blood.v82.8.2340.2340 8400286

[46] Eaves CJ, Eaves AC. Erythropoietin (Ep) dose-response curves for three classes of erythroid progenitors in normal human marrow and in patients with polycythemia vera. Blood. 1978;52(6):1196–210. doi: 10.1182/blood.v52.6.1196.bloodjournal5261196 719172

[47] Ziegler JG, Nichols NB. Optimum settings for automatic controllers. J Fluids Eng. 1942;64(8):759–65. doi: 10.1115/1.4019264

[48] Åström KJ, Hägglund T. Advanced PID control. International Society of Automation; 2006.

[49] Kitano H. Biological robustness. Nat Rev Genet. 2004;5(11):826–37. doi: 10.1038/nrg1471 15520792

[50] Szekely P, Sheftel H, Mayo A, Alon U. Evolutionary tradeoffs between economy and effectiveness in biological homeostasis systems. PLoS Comput Biol. 2013;9(8):e1003163. doi: 10.1371/journal.pcbi.1003163 23950698 PMC3738462

[51] Shoval O, Sheftel H, Shinar G, Hart Y, Ramote O, Mayo A, et al. Evolutionary trade-offs, Pareto optimality, and the geometry of phenotype space. Science. 2012;336(6085):1157–60. doi: 10.1126/science.1217405 22539553

[52] Ritchie ND, Ritchie R, Bayes HK, Mitchell TJ, Evans TJ. IL-17 can be protective or deleterious in murine pneumococcal pneumonia. PLoS Pathog. 2018;14(5):e1007099. doi: 10.1371/journal.ppat.1007099 29813133 PMC5993294

[53] Ndoricyimpaye EL, Van Snick J, Robert R, Bikorimana E, Majyambere O, Mukantwari E, et al. Cytokine kinetics during progression of COVID-19 in Rwanda patients: could IL-9/IFNγ ratio predict disease severity?. Int J Mol Sci. 2023;24(15):12272. doi: 10.3390/ijms241512272 37569646 PMC10418469

[54] Sharif-Askari FS, Sharif-Askari NS, Hafezi S, Mdkhana B, Alsayed HAH, Ansari AW, et al. Interleukin-17, a salivary biomarker for COVID-19 severity. PLoS One. 2022;17(9):e0274841. doi: 10.1371/journal.pone.0274841 36136963 PMC9498944

[55] Ahmed Mostafa G, Mohamed Ibrahim H, Al Sayed Shehab A, Mohamed Magdy S, AboAbdoun Soliman N, Fathy El-Sherif D. Up-regulated serum levels of interleukin (IL)-17A and IL-22 in Egyptian pediatric patients with COVID-19 and MIS-C: relation to the disease outcome. Cytokine. 2022;154:155870. doi: 10.1016/j.cyto.2022.155870 35398721 PMC8977483

[56] Yuan S, Jiang S-C, Zhang Z-W, Fu Y-F, Hu J, Li Z-L. Quantification of cytokine storms during virus infections. Front Immunol. 2021;12:659419. doi: 10.3389/fimmu.2021.659419 34079547 PMC8165266

[57] Mahallawi WH, Khabour OF, Zhang Q, Makhdoum HM, Suliman BA. MERS-CoV infection in humans is associated with a pro-inflammatory Th1 and Th17 cytokine profile. Cytokine. 2018;104:8–13. doi: 10.1016/j.cyto.2018.01.025 29414327 PMC7129230

[58] Fouquet G, Thongsa-Ad U, Lefevre C, Rousseau A, Tanhuad N, Khongkla E, et al. Iron-loaded transferrin potentiates erythropoietin effects on erythroblast proliferation and survival: a novel role through transferrin receptors. Exp Hematol. 2021;99:12-20.e3. doi: 10.1016/j.exphem.2021.05.005 34077792

[59] Coulon S, Dussiot M, Grapton D, Maciel TT, Wang PHM, Callens C, et al. Polymeric IgA1 controls erythroblast proliferation and accelerates erythropoiesis recovery in anemia. Nat Med. 2011;17(11):1456–65. doi: 10.1038/nm.2462 22019886

[60] Peslak SA, Wenger J, Bemis JC, Kingsley PD, Frame JM, Koniski AD, et al. Sublethal radiation injury uncovers a functional transition during erythroid maturation. Exp Hematol. 2011;39(4):434–45. doi: 10.1016/j.exphem.2011.01.010 21291953 PMC3523099

[61] Maira D, Duca L, Busti F, Consonni D, Salvatici M, Vianello A, et al. The role of hypoxia and inflammation in the regulation of iron metabolism and erythropoiesis in COVID-19: the IRONCOVID study. Am J Hematol. 2022;97(11):1404–12. doi: 10.1002/ajh.26679 36215667 PMC9538950

[62] Somers VK, Kara T, Xie J. Progressive hypoxia: a pivotal pathophysiologic mechanism of COVID-19 pneumonia. Mayo Clin Proc. 2020;95(11):2339–42. doi: 10.1016/j.mayocp.2020.09.015 33153625 PMC7524673

[63] Xie J, Covassin N, Fan Z, Singh P, Gao W, Li G, et al. Association between hypoxemia and mortality in patients with COVID-19. Mayo Clin Proc. 2020;95(6):1138–47. doi: 10.1016/j.mayocp.2020.04.006 32376101 PMC7151468

[64] Wu H, Klingmüller U, Acurio A, Hsiao JG, Lodish HF. Functional interaction of erythropoietin and stem cell factor receptors is essential for erythroid colony formation. Proc Natl Acad Sci U S A. 1997;94(5):1806–10. doi: 10.1073/pnas.94.5.1806 9050860 PMC19998

[65] Wessely O, Bauer A, Quang CT, Deiner EM, von Lindern M, Mellitzer G, et al. A novel way to induce erythroid progenitor self renewal: cooperation of c-Kit with the erythropoietin receptor. Biol Chem. 1999;380(2):187–202. doi: 10.1515/BC.1999.027 10195426

[66] Socolovsky M, Fallon AE, Lodish HF. The prolactin receptor rescues EpoR-/- erythroid progenitors and replaces EpoR in a synergistic interaction with c-kit. Blood. 1998;92(5):1491–6. doi: 10.1182/blood.v92.5.1491 9716574

[67] Broudy VC, Lin NL, Priestley GV, Nocka K, Wolf NS. Interaction of stem cell factor and its receptor c-kit mediates lodgment and acute expansion of hematopoietic cells in the murine spleen. Blood. 1996;88(1):75–81. doi: 10.1182/blood.v88.1.75.bloodjournal88175 8704204

[68] Kapur R, Zhang L. A novel mechanism of cooperation between c-Kit and erythropoietin receptor. Stem cell factor induces the expression of Stat5 and erythropoietin receptor, resulting in efficient proliferation and survival by erythropoietin. J Biol Chem. 2001;276(2):1099–106. 11042182 10.1074/jbc.M007442200

[69] Li K, Miller C, Hegde S, Wojchowski D. Roles for an Epo receptor Tyr-343 Stat5 pathway in proliferative co-signaling with kit. J Biol Chem. 2003;278(42):40702–9. doi: 10.1074/jbc.M307182200 12909618

[70] Zeuner A, Pedini F, Signore M, Testa U, Pelosi E, Peschle C, et al. Stem cell factor protects erythroid precursor cells from chemotherapeutic agents via up-regulation of BCL-2 family proteins. Blood. 2003;102(1):87–93. doi: 10.1182/blood-2002-08-2369 12637332

[71] von Lindern M, Schmidt U, Beug H. Control of erythropoiesis by erythropoietin and stem cell factor: a novel role for Bruton’s tyrosine kinase. Cell Cycle. 2004;3(7):876–9. doi: 10.4161/cc.3.7.1001 15254422

[72] Gregory CJ, McCulloch EA, Till JE. Transient erythropoietic spleen colonies: effects of erythropoietin in normal and genetically anemic W/Wv mice. J Cell Physiol. 1975;86(1):1–8. doi: 10.1002/jcp.1040860102 1176537

[73] Nocka K, Tan JC, Chiu E, Chu TY, Ray P, Traktman P, et al. Molecular bases of dominant negative and loss of function mutations at the murine c-kit/white spotting locus: W37, Wv, W41 and W. EMBO J. 1990;9(6):1805–13. doi: 10.1002/j.1460-2075.1990.tb08305.x 1693331 PMC551885

[74] Schwarzenberger P, Huang W, Ye P, Oliver P, Manuel M, Zhang Z, et al. Requirement of endogenous stem cell factor and granulocyte-colony-stimulating factor for IL-17-mediated granulopoiesis. J Immunol. 2000;164(9):4783–9. doi: 10.4049/jimmunol.164.9.4783 10779785

[75] Tan W, Liu B, Barsoum A, Huang W, Kolls JK, Schwarzenberger P. Requirement of TPO/c-mpl for IL-17A-induced granulopoiesis and megakaryopoiesis. J Leukoc Biol. 2013;94(6):1303–8. doi: 10.1189/jlb.1212639 23990627 PMC4051276

[76] Song X, Dai D, He X, Zhu S, Yao Y, Gao H, et al. Growth factor FGF2 cooperates with Interleukin-17 to repair intestinal epithelial damage. Immunity. 2015;43(3):488–501. doi: 10.1016/j.immuni.2015.06.024 26320657

[77] Weiss G, Ganz T, Goodnough LT. Anemia of inflammation. Blood. 2019;133(1):40–50. doi: 10.1182/blood-2018-06-856500 30401705 PMC6536698

[78] Canny SP, Orozco SL, Thulin NK, Hamerman JA. Immune mechanisms in inflammatory anemia. Annu Rev Immunol. 2023;41:405–29. doi: 10.1146/annurev-immunol-101320-125839 36750316 PMC10367595

[79] Roelli P, bbimber, Flynn B, Santiagorevale, Gui G. Hoohm/CITE-seq-Count: 1.4.2. 2019.

[80] McGinnis CS, Patterson DM, Winkler J, Conrad DN, Hein MY, Srivastava V, et al. MULTI-seq: sample multiplexing for single-cell RNA sequencing using lipid-tagged indices. Nat Methods. 2019;16(7):619–26. doi: 10.1038/s41592-019-0433-8 31209384 PMC6837808

[81] Tabula Muris Consortium, Overall coordination, Logistical coordination, Organ collection and processing, Library preparation and sequencing, Computational data analysis, et al. Single-cell transcriptomics of 20 mouse organs creates a Tabula Muris. Nature. 2018;562(7727):367–72. doi: 10.1038/s41586-018-0590-4 30283141 PMC6642641

[82] Kimmel JC, Penland L, Rubinstein ND, Hendrickson DG, Kelley DR, Rosenthal AZ. Murine single-cell RNA-seq reveals cell-identity- and tissue-specific trajectories of aging. Genome Res. 2019;29(12):2088–103. doi: 10.1101/gr.253880.119 31754020 PMC6886498

[83] Whitfield ML, Sherlock G, Saldanha AJ, Murray JI, Ball CA, Alexander KE, et al. Identification of genes periodically expressed in the human cell cycle and their expression in tumors. Mol Biol Cell. 2002;13(6):1977–2000. doi: 10.1091/mbc.02-02-0030 12058064 PMC117619

[84] Subramanian A, Tamayo P, Mootha VK, Mukherjee S, Ebert BL, Gillette MA, et al. Gene set enrichment analysis: a knowledge-based approach for interpreting genome-wide expression profiles. Proc Natl Acad Sci U S A. 2005;102(43):15545–50. doi: 10.1073/pnas.0506580102 16199517 PMC1239896

[85] Tirosh I, Izar B, Prakadan SM, Wadsworth MH 2nd, Treacy D, Trombetta JJ, et al. Dissecting the multicellular ecosystem of metastatic melanoma by single-cell RNA-seq. Science. 2016;352(6282):189–96. doi: 10.1126/science.aad0501 27124452 PMC4944528

