# Supplementary figures and images for: IL-17 stimulates erythropoiesis in vivo by amplifying the response of erythroid progenitors to erythropoietin
Source: PLoS Biol. 2025 Dec 11;23(12):e3003462. doi: 10.1371/journal.pbio.3003462 (PMC12697970; doi:10.1371/journal.pbio.3003462)

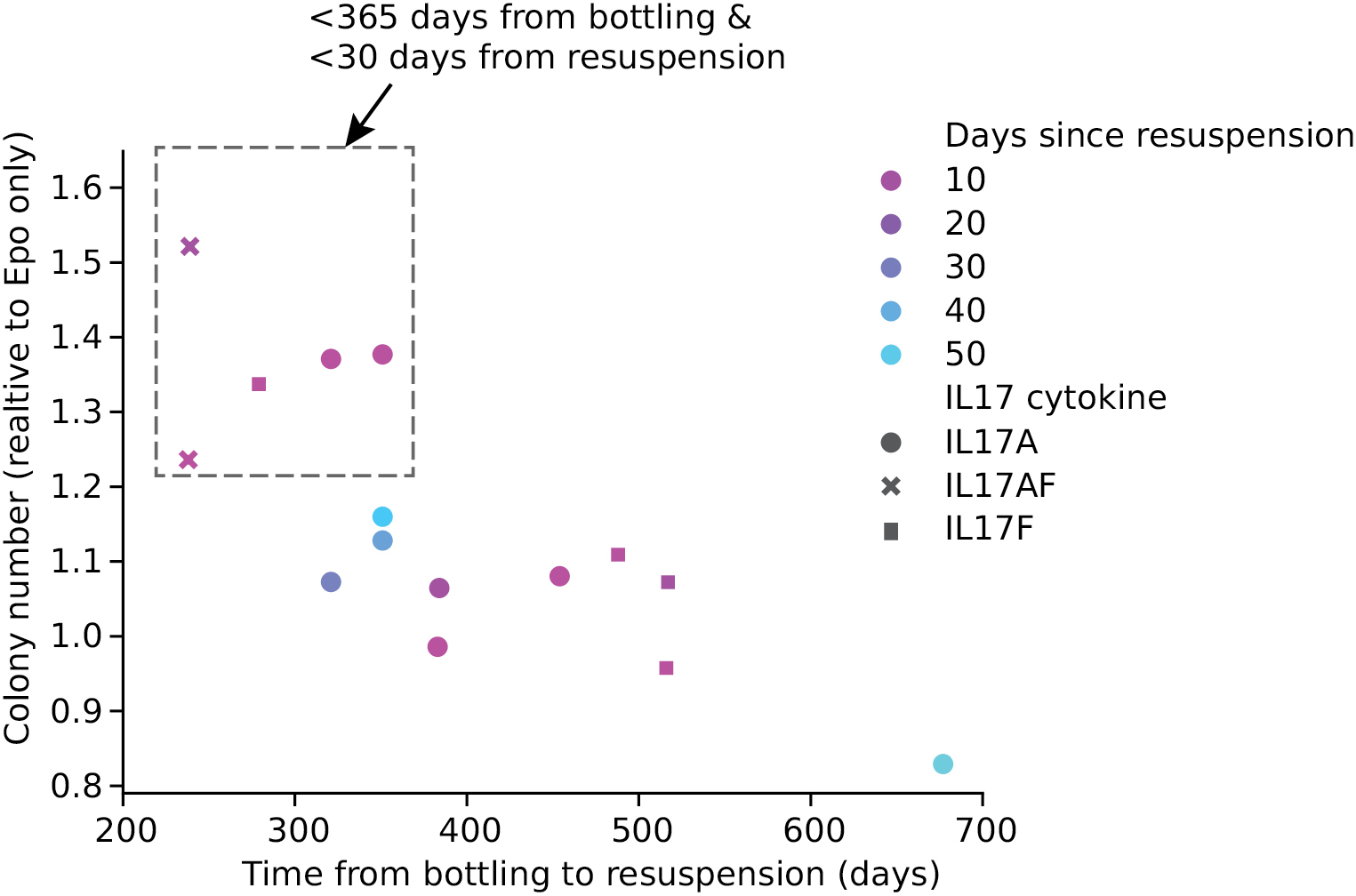

Supplement: S1 Fig — CFU-E assays were carried out as in Fig 1B Fig 1E. Lyophilized protein was purchased from R&D Systems. The time interval between bottling of the lyophilized protein by the manufacturer, and its resuspension prior to use, varied for different protein lots. Activity is lost if the lyophilized protein was bottled >365 days prior to the day of resuspension. In addition, once the protein is resuspended, its activity declined rapidly after 30 days. All IL-17 protein ligands (both lyophilized and resuspended) were stored at −80 °C. Data for this figure are in S1 Table. (TIF) [file pbio.3003462.s001.tif]

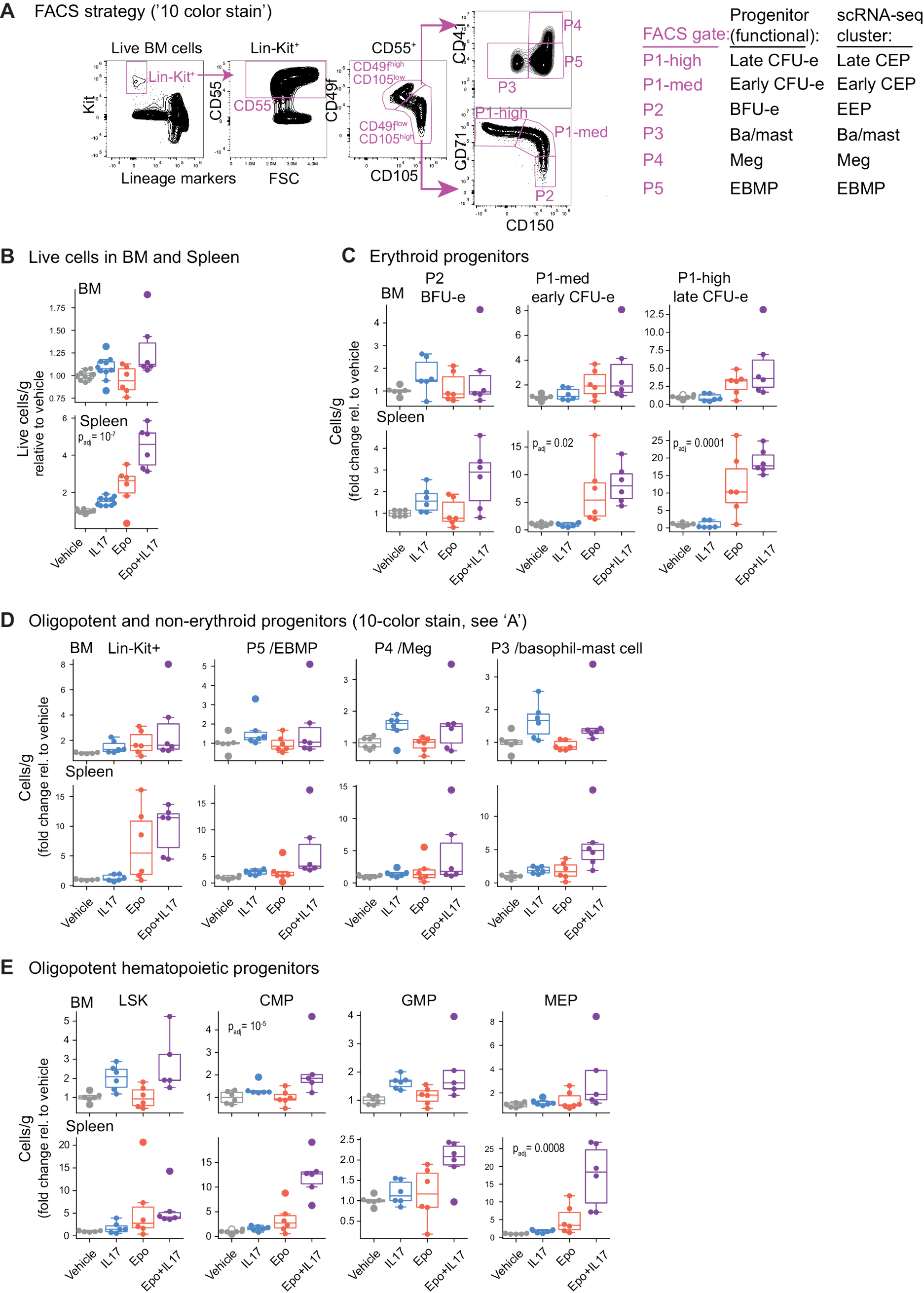

Supplement: S3 Fig — (A) FACS gating strategy for identifying early hematopoietic and erythroid progenitors [5,30] (the “10 color stain”). Corresponding FACS gates, functionally defined progenitors, and single-cell RNA-seq (scRNA-seq) clusters are shown on the right. Correspondence between the three modalities of defining each progenitor type was previously shown in Tusi and colleagues [5]. (B–E) Data underlying Fig 2F, broken out by mouse. (B–D) Summary data of bone marrow (BM) and spleen cells labeled with the ‘10 color stain’ antibody panel. Data points correspond to individual mice and box plots are defined as in Fig 2B. (E) Summary data of BM and spleen cells labeled with CD34, CD16/32 antibodies. CMP, common myeloid progenitor; MEP, megakaryocytic/erythrocytic progenitor; GMP, granulocytic/monocytic progenitor. Data for panels A–E are in S2 Table. (TIF) [file pbio.3003462.s003.tif]

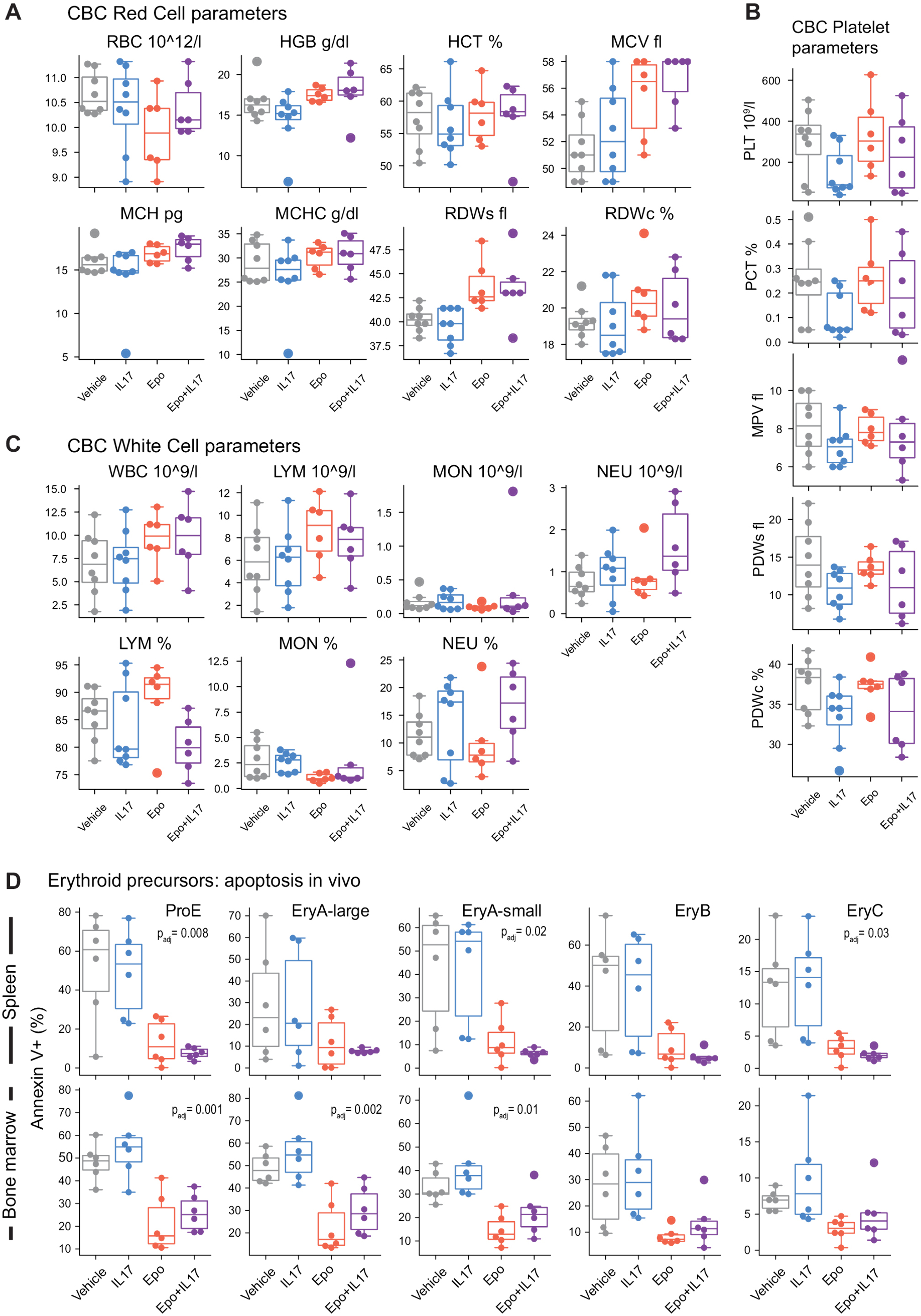

Supplement: S4 Fig — (A–C) CBC results from experiments described in Fig 2. Blood was obtained by cardiac puncture immediately following culling. Data points correspond to individual mice and box plots are defined as in Fig 2B. (D) Flow-cytometric analysis of apoptosis in erythroid precursors in vivo. Annexin V labeling of cells in each of the erythroid subsets shown in S2B Fig. Data for panels A–D are in S2 Table. (TIF) [file pbio.3003462.s004.tif]

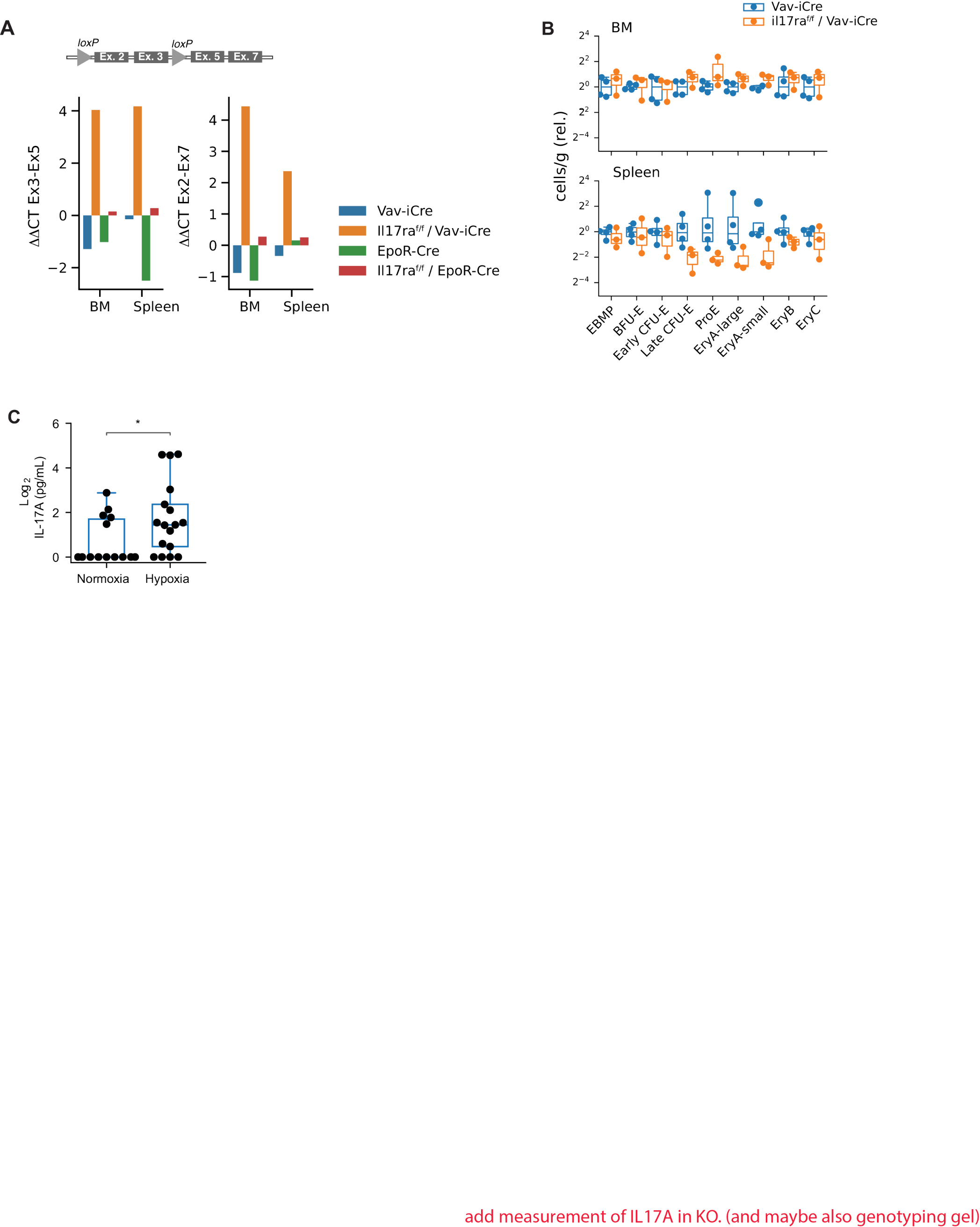

Supplement: S5 Fig — (A) qPCR analysis of Il17ra deletion assessed using whole bone marrow or whole spleen. Two alternative primer sets gave similar results: either primers to exons 3 and 5, or exons 2 and 7. For Vav-iCre-mediated deletion, deletion efficiency in whole tissue was less efficient than in sorted hematopoietic cells (either Kit+ or ProE), see Fig 3. EpoR-Cre-mediated deletion was poor. (B) Erythroid progenitors and precursors in Vav-iCre and Il17raf/f/ Vav-iCre, analyzed by flow cytometry using CD71/Ter119 and the “10 color stain” panels (see S3 Fig). (C) Measured blood serum concentrations of IL-17A in mice in normoxia and following 24 hours of exposure to hypoxia following the treatment schema in Fig 3D. Measurements are carried out by immunoPCR along with recombinant murine IL-17A as a standard curve. Data points correspond to individual mice, and box plots are defined as in Fig 2B. The increase in IL-17A in hypoxia is statistically significant (Wilcoxon rank-sum test, one-tailed p-value <0.05) but still well below the EC50 of erythroid progenitors seen in CFU-E assays. Data for all panels are in S12 Table. (TIF) [file pbio.3003462.s005.tif]

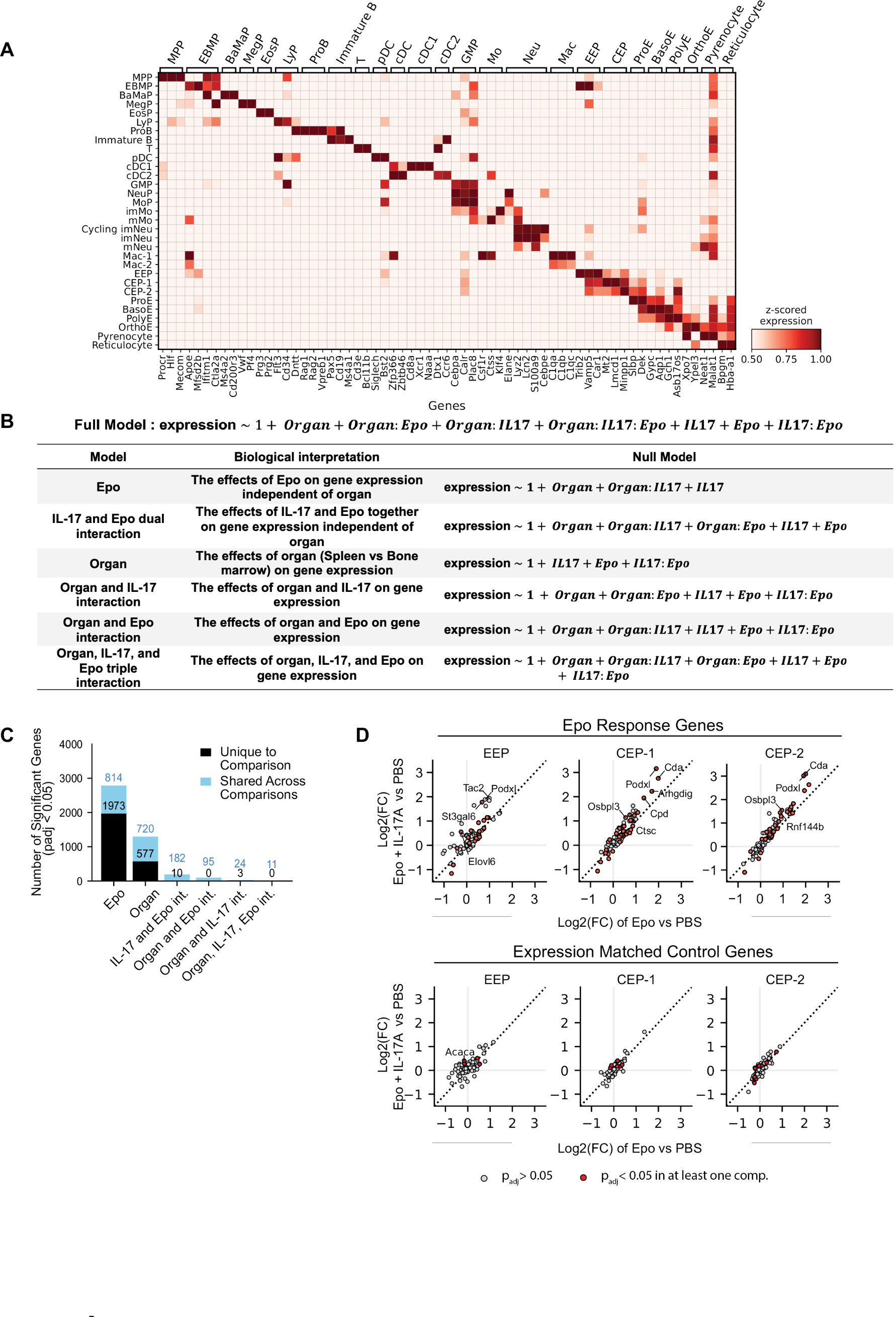

Supplement: S6 Fig — (A) Heatmap showing expression of cell type-specific marker genes (columns) across cell clusters annotated to different hematopoietic cell states (rows). The genes were selected from literature as markers of cell states, as shown by their group label at the top. Marker gene list and the corresponding origins can be found in S5 Table. The diagonal pattern demonstrates marker gene specificity for their respective cell populations. Expression values are the mean of z-score standardized values. (B) Definition of the generalized linear model (GLM) and the nested null models used to dissect gene expression dependency on cytokine treatments and tissue/organ-of-origin for each gene, supporting Fig 4F. The full model incorporates all possible interactions between organ/tissue (spleen versus bone marrow), Epo, and IL-17A treatments. The nested models remove terms to enable statistical significance testing for different terms (Likelihood Ratio Tests followed by multiple hypothesis correction across all genes, see Methods). The biological interpretation and corresponding null model formula are shown for each comparison. (C) Distribution of significantly regulated genes (padj < 0.01, |log2FC| > 0.25) from applying the GLM to CEP-1. The six bars correspond to testing against each of the null models tabulated in (B). Black bars indicate genes uniquely regulated in a single comparison, while blue bars show genes shared across multiple comparisons. The analysis reveals that Epo treatment and tissue-specific differences are the dominant sources of transcriptional variation. The IL-17A and Epo interaction showed limited unique gene regulation (10 unique genes), with most regulated genes shared with other comparisons. Similar distributions were observed across other erythroid populations. The number of genes which are overlapping between Epo treatment and IL-17A and Epo interaction are shown in Fig 4F.D Top: comparison of changes in the expression of Epo response genes across erythroid [file pbio.3003462.s006.tif]

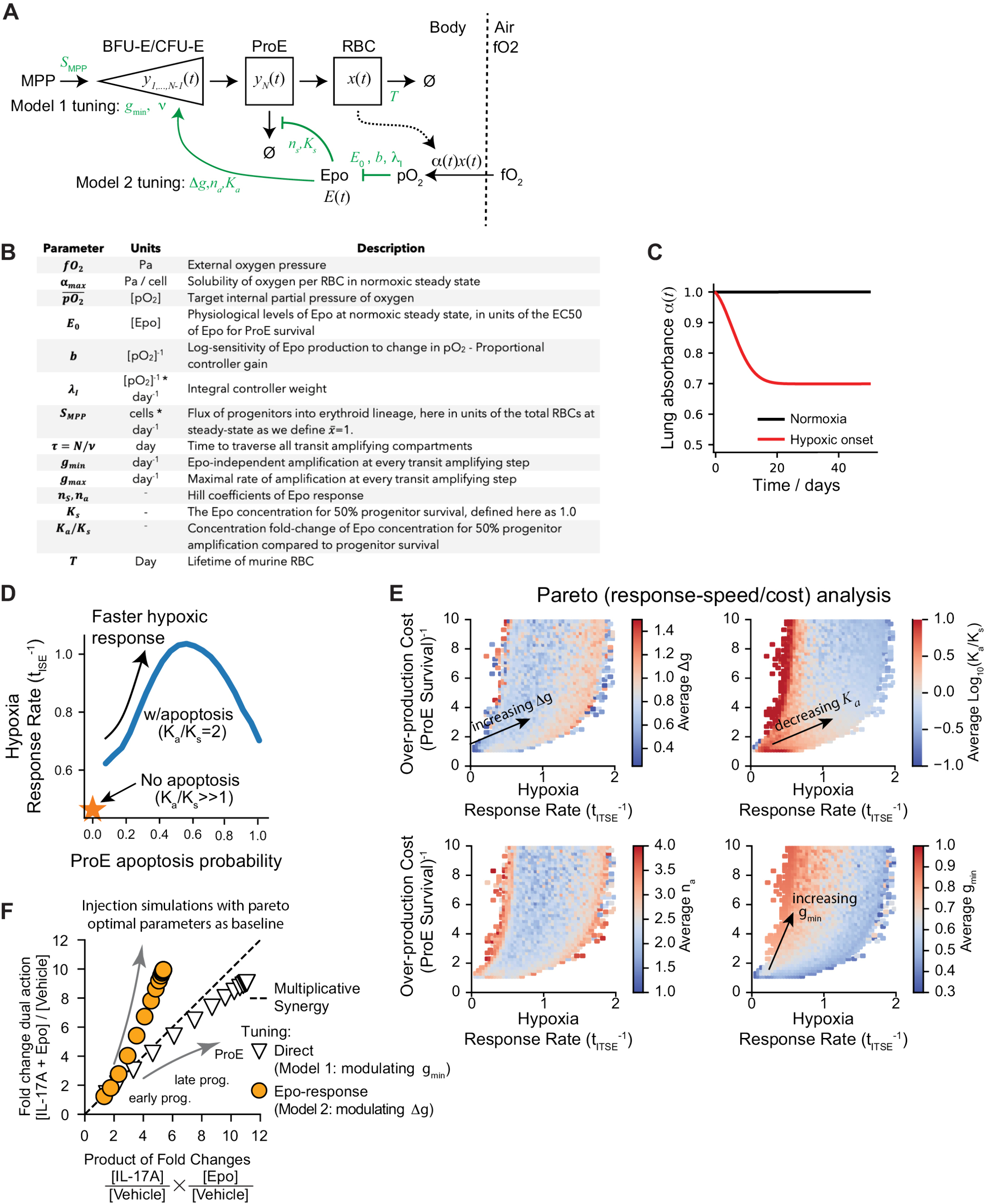

Supplement: S7 Fig — (A) Diagram of the dynamical systems model of erythropoietic feedback control. Black arrows indicate cell flux. Green arrows indicate regulatory links. Symbols in green show parameters associated with different model components. The full mathematical model is defined in S1 Text. (B) Table defining the model parameters in (A). (C) The dynamics of lung absorbance used to model in disease-associated hypoxic onset in Fig 7 and all subsequent analyses (Fig 7D–7F, and panels 7D, 7E below). The graphs show the value of model parameter for oxygen absorbance through the lung, α(t), as defined in S1 Text (Eq. [10]). (D) The model recapitulates a requirement for ProE apoptosis in accelerating hypoxic response rates, defined as in Fig 7C. The orange star denotes the response rate when Ka/Ks≫1, gmin=0.3/day,Δg=0.9/day, corresponding to a case where all ProE survive and Epo exclusively regulates cell proliferation. The blue curve corresponds to the same Epo regulation of progenitors, but now with Ka/Ks=2.0 corresponding to Epo regulating both response rate and survival. Variation along the blue curve represents varying values of the Epo-independent proliferation rate gmin, with higher values leading to high apoptosis rates. (E) Random sampling of model parameters in 105 simulations reveal different trade-offs in overproduction cost and response rate across parameters. This panel extends Fig 7F. Parameters were sampled over the intervals: gmin∈[0.3,1.3]/day, Δg∈[0.3,1.3]/day, Ka/Ks∈[0.05,20], na∈[1,3]. (F) Computational predictions for the expansion of erythroid progenitors in each of the two Models following Epo + IL-17A treatment as compared to multiplicative fold change of Epo and IL-17A alone. Pareto optimal parameters were used to define baseline. The action of model 2 is defined here as modulation of Δg. The dashed line indicates multiplicative synergy. Refer to S1 Text for modeling parameters used. The data for panels C–F are in S12 Table. (TIF) [file pbio.3003462.s007.tif]
