# Supplementary material for: IL-17 stimulates erythropoiesis in vivo by amplifying the response of erythroid progenitors to erythropoietin
Source: PLoS Biol. 2025 Dec 11;23(12):e3003462. doi: 10.1371/journal.pbio.3003462 (PMC12697970; doi:10.1371/journal.pbio.3003462)
Supplement: S1 Text — Definition of the dynamical systems model shown in Figs 7 and S7, including details on generation of all modeling figure panels. (DOCX) [file pbio.3003462.s008.docx]

**Supplemental text: Dynamical modeling of a burden/response trade-off in erythropoiesis**

This supplement defines the dynamical model used to evaluate alternative hypotheses for homeostatic control tuning of red blood cell (RBC) production, supporting **Figs. 7** and **S7**.

**1. Model Definition**

We adopt a dynamical systems model of erythropoiesis represented by a series of ODEs and algebraic equations describing system variables. Several prior ODE models of erythropoiesis have been developed^1^ that incorporate a considerably greater level of detail than described here, but these lacked the control elements of the current model.

*1.1 Model design overview.*

The model is designed to capture the relevant biological processes that effect a trade-off between two performance goals of the erythropoietic homeostatic control circuit, and which are likely under control of an early-expressed receptor: (i) maximizing the speed of recovery from hypoxic stress, and (ii) minimizing the burden of constitutive progenitor cell over-production in normoxia. This trade-off results from two key features of RBC production:

1. A lag time in differentiation and the transit amplification of erythroid progenitors^2,3^.
2. A homeostatic negative feedback circuit: red blood cells (RBCs) elevate oxygen blood concentration (pO₂); pO₂ represses Epo production; and Epo stimulates RBC production ^4,5^.

We incorporate two specific the mechanisms of Epo action on erythropoiesis: (1) Epo prevents death of later precursors (proerythroblasts, or ProE) ^6–8^ ; and (2) Epo increases proliferation in progenitors^9,10^ leading to increased reticulocyte production in 5-7 days^11,12^, but does so at lower affinity.

The model does not incorporate many more detailed features of erythropoiesis, which we reasoned would add to complexity without qualitatively altering the key predictions that differentiate hypotheses regarding IL-17A function evaluated in the text. Notably, we ignore: (1) proliferation subsequent to ProE; (2) distinction between reticulocytes and RBCs; (3) nomenclature of early progenitors; (4) Epo uptake by erythroid progenitors; (5) density-dependent Epo responses mediated by Fas/FasL^10^. In addition, (6) we have not explicitly accounted for other effects of elevated Epo concentrations on erythropoiesis. Epo stimulates the release of premature reticulocytes into blood, offering a short-term response to hypoxic stress in <1 day ^13,14^. It additionally promotes division of late erythroid progenitors (EryA/B). These effects will quantitatively accelerate hypoxic responses, and all reducing resting costs and buffer the lag in generating new cells through progenitor division. However, they should do not qualitatively change RBC response profiles over longer periods (>3 days in mice, or >5-7 days in humans). The discriminating features related to IL-17A action discussed in this paper relate to longer latency periods and we reasoned that they can be described without incorporating the late-stage behaviors.

*1.2 Model variables.*

The model, shown schematically in **Figs. 7A and S7A** has the following dynamic variables:

| Variable | Units | Description |
| --- | --- | --- |
| $\boldsymbol{p}\boldsymbol{O}_{\boldsymbol{2}}\left( \boldsymbol{t} \right)$ | Pa | Partial pressure of oxygen (in blood) |
| *E*$\left( \boldsymbol{t} \right)$ | - | Erythropoietin concentration, in units of the EC50 for ProE survival |
| $\boldsymbol{y}_{\boldsymbol{1,\ldots,N-1}}\left( \boldsymbol{t} \right)$ | # cells | Number of progenitor cells along *N-1* steps of differentiation |
| $\boldsymbol{y}_{\boldsymbol{N}}\left( \boldsymbol{t} \right)$ | # cells | Number of ProE cells |
| *x*$\left( \boldsymbol{t} \right)$ | # cells | Number RBCs |

These variables include *N* numerically labeled stages of progenitor differentiation, where a choice of *N* is used to limit the variance in the latency time of transit amplification. The choice of *N* is not intended to reflect specific biological transitions, although one may think of BFU-E/EEP as reflecting early progenitors $y_{1,\ldots,n<N-1}$, and CFU-E/CEP as reflecting the later progenitors $y_{n+1,\ldots,N-1}$. Variation in *N* is not expected to alter model conclusions, provided $N\gg1$. We have used *N*=20. A more precise definition of “large N” is given in section 3 below. For the current analysis, the transition stage *n* between EEP and CEP plays has no role and is not discussed further.

*1.3 Model equations and parameters*

The model architecture is shown schematically in **Fig. S7A** and is fully defined in the following sub-sections. The model parameters are non-dimensionalized where possible as described in the tables below.

*1.3.1 Fast variables: oxygen and erythropoietin.*

We assume that oxygen and Epo concentrations ($pO_{2}\left( t \right)$, $E\left( t \right)$) are in quasi-steady-state as they equilibrate rapidly compared to the timescale of cell differentiation in erythropoiesis. Oxygen equilibration occurs within minutes through pulmonary gas exchange. Epo half-life in the blood is ~12 hours. By contrast, the dynamics of cell populations responds over days.

In our model the oxygen dissolved in blood is assumed to be directly proportional to the RBC mass, i.e.:

$$\text{[1] }pO_{2}\left( t \right)=fO_{2}\cdot\alpha\left( t \right)\cdot x\left( t \right)$$

where here $fO_{2}$ is the environmental oxygen pressure, and $\alpha\left( t \right)$ is the solubility of oxygen per RBC at time *t*. In steady-state healthy conditions, we assume $\alpha\left( t \right)=\alpha_{max}$. Simulations of infection-induced hypoxic stress are carried out by providing a dynamic value of $\alpha\left( t \right)$ that drops from $\alpha_{max}$ over time to a lower value, as discussed below. A more detailed model of oxygen transport would address changes in the cooperativity of hemoglobin as a function of blood pH, as well as changes in hematocrit. Both vary with hypoxia. However, in this model we make a simplification by ignoring cooperativity of binding and treated RBC mass rather than blood concentration.

For Epo, production is generated in response to hypoxia by cells in the kidney and we take it to be:

$$\left[ 2 \right] E\left( t \right)=E_{0}\exp\left[ -b\left( pO_{2}\left( t \right)-\bar{pO_{2}} \right) \right]-\lambda_{I}\int_{-\infty}^{t} \left( pO_{2}\left( t^{'} \right)-\bar{pO_{2}} \right)dt^{'}$$

Here, the first term reflects proportional control of Epo production in the kidney in response to changes in oxygen tension. Eq. [2] is defined such that $E\left( t \right)=E_{0}$ at steady-state. The concentration of Epo mRNA has been shown to grow exponentially with the drop in partial pressure, which is reflected in the exponential factor with sensitivity to oxygen changes $b$. In addition, we include a second term reflecting an integral feedback controller with strength $\lambda_{I}$ [Eqn. 2]. Integral feedback control ensures robustness of the steady-state to fluctuations in system parameters. In summary, the table below summarizes the parameters included in this sub-section:

| Parameter | Units | Description |
| --- | --- | --- |
| $\boldsymbol{f}\boldsymbol{O}_{\boldsymbol{2}}$ | Pa | External oxygen pressure |
| $\boldsymbol{\alpha}_{\boldsymbol{max}}$ | Pa / cell | Solubility of oxygen per RBC in normoxic steady state |
| $\bar{\boldsymbol{p}\boldsymbol{O}_{\boldsymbol{2}}}$ | [pO_2_] | Target internal partial pressure of oxygen |
| $\boldsymbol{E}_{\boldsymbol{0}}$ | [Epo] | Physiological levels of Epo at normoxic steady state, in units of the EC50 of Epo for ProE survival |
| $\boldsymbol{b}$ | [pO_2_]^-1^ | Log-sensitivity of Epo production to change in pO_2_ - Proportional controller gain |
| $\boldsymbol{\lambda}_{\boldsymbol{I}}$ | [pO_2_]^-1^ * day^-1^ | Integral controller weight |

*1.3.2 Slow variables: erythroid progenitors*

**Figs. 7A, S7A** show schematically the differentiation hierarchy from multipotent progenitors to RBCs. This hierarchy is model through a set of coupled linear ODEs:

[3] $\text{ }\dot{y_{1}}\left( t \right)=S_{MPP}-\left[ \nu-g\left( E\left( t \right) \right) \right]y_{1}\left( t \right)$

[4] $\dot{y_{k}}\left( t \right)=\nu\cdot y_{k-1}\left( t \right)-\left[ \nu-g\left( E\left( t \right) \right) \right]y_{k}\left( t \right) \text{for }\text{k}\text{=[2,…,}\text{N}\text{]}$

[5] $\dot{x}\left( t \right)=\nu\cdot H\left( E\left( t \right),n_{s},K_{s} \right)\cdot y_{N}\left( t \right)-x\left( t \right)/T$

Here, Eqs. [3] and [4] describe the dynamics of the BFU-E and CFU-E cells progressing through stages of differentiation, and Eq. [5] describes dynamics of the RBCs.

Eq. [3] describes dynamics of the earliest erythroid-committed progenitors [compartment $y_{1}$], which arise from progenitors (MPPs) that are not under control of Epo. The rate of MPP differentiation is thus treated as constant in the model, defined by the rate parameter $S_{MPP}$. In both Eqs. [3] and [4], progenitors in each compartment $y_{k}$ differentiate to the subsequent compartment $y_{k+1}$ with mean rate $\nu$. The progenitors proliferate with an Epo-dependent rate $g\left( E \right)$, given by

[6] $g\left( E\left( t \right) \right)=g_{min}+\left( g_{max}-g_{min} \right)H\left( E\left( t \right),n_{a},K_{a} \right)$].

where $g_{min}$ is the Epo-independent early progenitor proliferation rate, $g_{max}$ is the maximal proliferation rate at saturating Epo concentrations, and $H\left( z,n,K \right)=z^{n}/\left[ K^{n}+z^{n} \right]$ is a Hill function with coefficient *n* and half-max concentration *K*.

The dynamics of RBCs (Eq. [5]) incorporates the final elements of the model: the first term in this equation describes ProE cells [compartment $y_{N}$] differentiating into RBCs at a rate $\nu$, but with only a fraction $H\left( E\left( t \right),n_{s},K_{s} \right)$ of the cells surviving in an Epo-dependent manner. Here *H* is a Hill function defined as above, but with a different half-max concentration $K_{s}$ for ProE survival and coefficient *n_s_*. RBCs are cleared with an average lifetime *T*. As discussed in subsection 1.1, this model ignores cell division after ProE formation and does not distinguish reticulocytes from RBCs.

The model parameters in Eqs. [3-6] are summarized in the following table, along with the values used for simulation.

| Parameter | Units | Description |
| --- | --- | --- |
| $\boldsymbol{S}_{\boldsymbol{MPP}}$ | cells * day^-1^ | Flux of progenitors into erythroid lineage, here in units of the total RBCs at steady-state as we define $\bar{x}$=1. |
| $\boldsymbol{\tau=N}\boldsymbol{/}\boldsymbol{\nu}$ | day | Time to traverse all transit amplifying compartments |
| $\boldsymbol{g}_{\boldsymbol{min}}$ | day^-1^ | Epo-independent amplification at every transit amplifying step |
| $\boldsymbol{g}_{\boldsymbol{max}}$ | day^-1^ | Maximal rate of amplification at every transit amplifying step |
| $\boldsymbol{n}_{\boldsymbol{S}}\boldsymbol{,}\boldsymbol{n}_{\boldsymbol{a}}$ | – | Hill coefficients of Epo response |
| $\boldsymbol{K}_{\boldsymbol{s}}$ | - | The Epo concentration for 50% progenitor survival, defined here as 1.0 |
| $\boldsymbol{K}_{\boldsymbol{a}}\boldsymbol{/}\boldsymbol{K}_{\boldsymbol{s}}$ | – | Concentration fold-change of Epo concentration for 50% progenitor amplification compared to progenitor survival |
| $\boldsymbol{T}$ | Day | Lifetime of murine RBC |

**2. Normoxic steady state and its constitutive over-production cost.**

The over-production of ProE in steady state normoxic conditions represents a constitutive cost of the model. We define the over-production burden as the ratio of produced ProE cells to those surviving, i.e., cost $\theta_{c}$

[7] $\theta_{c}=1/H\left( \bar{E},n,K_{s} \right)$.

To calculate this cost for a given parameter choice, we solve the dynamical equations at steady state with ${\alpha\left( t \right)=\alpha}_{max}=1$. We denote steady-state values of the dynamical system variables with an over-bar ($\bar{x},\bar{y}_{k},$ etc). At steady-state, Eq. [2] enforces $pO_{2}\left( t \right)=\bar{pO_{2}}$, and so from Eq. [1] the steady-state RBC mass is $\bar{x}=\frac{\bar{pO_{2}}}{\alpha_{max} fO_{2}}.$ A convenient choice of units sets $\bar{x}=1$ as discussed in section 3 below.

We now provide the steady-state solutions for the cell populations $\bar{y}_{k}$ from Eqs. [3-6]:

[8] $\bar{y}_{k}=\frac{S_{MPP}}{\nu}\frac{1}{\left( 1-g\left( \bar{E} \right)/\nu\right)^{k}}\text{ for }k=\left[ 1,\ldots,N \right]$

[9] $\bar{x}\equiv1=S_{MPP}T\frac{1}{\left( 1-g\left( \bar{E} \right)/\nu\right)^{N}}\cdot H\left( \bar{E},n,K_{s} \right)$

A steady-state is achieved when the parameters thus satisfy Eq. [9]. When *N* is large, $\frac{1}{\left( 1-g\left( \bar{E} \right)/\nu\right)^{N}}\approx e^{g\left( \bar{E} \right)\tau}$ and is independent of *N*. Note that $\bar{E}$ only enters the steady-state equations through its EC50 values $\bar{E}/K_{s}$, $\bar{E}/K_{a}$. Its value is not determined by Eq. [2] because the integral control term allows adding an arbitrary offset $\bar{E}=E_{0}+\delta E$, e.g. $\delta E=\lambda_{I}{\int_{-\infty}^{0} \left( pO_{2}\left( t^{'} \right)-\bar{pO_{2}} \right)dt}^{'}.$

In this study, a numerical solver was used in Python to solve Eq. [8] for $\bar{E}$ once remaining model parameters were established. For simplicity, we set $E_{0}=\bar{E}$ for a given choice of parameters. Subsequently, the over-production cost $\theta_{c}$ can be calculated from Eq. [9].

**3. Parameter values**

Parameter values used in simulation are defined in the tables above, with justification provided here. Without loss of generality, we have set units of oxygen pressure and cell number such that:

$\bar{pO_{2}}=fO_{2}=1, \alpha_{max}=1, \bar{x}=1,$ and $K_{s}=1$.

And $E_{0}$ is set by solving the steady-state equation Eq. [9] as described above. For the remaining parameters, we established values as follows.

1. The lifetime of RBCs in mice has been estimated several times in the literature with broadly consistent results. We have used the value of *T=*40 days (see reference in table above).
2. The time for mouse BFU-Es to give rise to colonies containing RBC is ~7 days, and so we have set $\tau=N/\nu=7$ days.
3. The choice of *N* is constrained by two technical aspects of the ODE model. We have used a choice of $N=20$. We motivate our choice as follows.
   1. In Eq. [9], as noted the solution is independent of *N* when *N* is large, so the precise value is not important.
   2. In an ODE modeling framework, the time taken to transition through a chain of *N* steps with equal transition rates $\nu$ follows a gamma distribution $t\sim\mathrm{Gamma}\left[ N,\frac{1}{\nu} \right]$. As such *N* sets the variability in the time of transit amplification. One notes that for the gamma distribution the mean time is $E\left[ t \right]=\frac{N}{\nu}=\tau$, and standard deviation is $\sigma_{t}=\tau/\sqrt{N}$. Therefore, *N* may be thought of as a convenience variable used to tune the sharpness of the latency time of the model, with a coefficient of variation $\frac{\sigma_{t}}{E\left[ t \right]}=1/\sqrt{N}$.
4. From Eq. [9] and assumptions above, we note that $A\approx e^{g\left( \bar{E} \right)\tau}$ corresponds to the total amplification that progenitors undergo. The precise number of cell divisions that progenitors undergo is not measured but is thought to be ~10. We have chosen an amplification of $A=1000$. From this we can constrain $g\left( \bar{E} \right)\sim0.8$/day, which sets upper and lower bounds on $g_{min}$ and $g_{max}$. This estimate is also in agreement with prior measurements of the mean cell division time for BFU-E/CFU-E in the fetal liver of mice, which is ~15 hours. The doubling time is $\frac{\ln\left( 2 \right)}{\left( \frac{15}{24} \right)\mathrm{day}}=1.1$/day.
5. Only one parameter remains that governs the steady-state behavior: the rate of cells entering the erythroid lineage from the MPP pool $S_{MPP}$. From Eq. [9] and assumptions 1-3,5 above, we can relate these parameters $S_{MPP}=\left( 1-g_{min}\tau/N \right)^{-N}\theta_{c}/T$. We note that $\left( 1-g_{min}\tau/N \right)^{-N}=A$ corresponds to the total amplification that progenitors undergo as defined in assumption 4 above. Thus, $S_{MPP}=\bar{x}/\left( AT \right)$ is fully specified.
6. We assume that, in the absence of IL-17A, the early progenitors are less responsive to Epo and thus $K_{a}>K_{s}$. (Note that $K_{s}=1$ sets the units of Epo, as discussed above).

This now leaves three parameters unconstrained but bounded: $K_{a}/K_{s}$, $g_{min}$, $g_{max}$. The results given in the paper hold for different choices of these three parameters. As a baseline, the following values were used:

**Table I. Baseline parameter values**

| Parameter | Value | Estimate Range | Reference |
| --- | --- | --- | --- |
| $\bar{\boldsymbol{p}\boldsymbol{O}_{\boldsymbol{2}}}$ | 1.0 | Units set to 1.0 | - |
| $\boldsymbol{f}\boldsymbol{O}_{\boldsymbol{2}}$ | 1.0 | Units set to 1.0 | - |
| $\boldsymbol{E}_{\boldsymbol{0}}$ | Set by Eq. [9] | – | - |
| $\boldsymbol{\alpha}_{\boldsymbol{max}}$ | 1.0 | – | - |
| $\boldsymbol{S}_{\boldsymbol{MPP}}$ | $1.25\cdot{10}^{-4}$ / day | – | - |
| $\boldsymbol{\tau}$ | 7.0 days | 7 Days | 12,15 |
| $\boldsymbol{g}_{\boldsymbol{min}}$ | 0.5/day |  | - |
| $\boldsymbol{g}_{\boldsymbol{max}}$ | 0.8/day | 1-3/day | - |
| $\boldsymbol{n}_{\boldsymbol{a}}\boldsymbol{,}\boldsymbol{n}_{\boldsymbol{s}}$ | 2.0 | 1-3 | 16 |
| $\boldsymbol{K}_{\boldsymbol{s}}$ | 1.0 | Units set to 1.0 | - |
| $\boldsymbol{K}_{\boldsymbol{a}}\boldsymbol{/}\boldsymbol{K}_{\boldsymbol{s}}$ | 1.5 | Lower Epo sensitivity in progenitors than ProE | 17,18 |
| $\boldsymbol{T}$ | 40.0 days | 40±1.9 days | 19 |
| $\boldsymbol{b}$ | 5 |  |  |
| $\boldsymbol{\lambda}_{\boldsymbol{I}}$ | 0.05 |  |  |

**4. Model Simulations.**

*4.1 Simulations* (**Figs. 7E and 7F, S7C-E**).

The dynamical equations were implemented and solved using Python v3.10.13, with the code available at github.com/AllonKleinLab/Wu2024. We used the integrate.solve_ivp function from SciPy v1.11.4, applying the 'BDF' method to solve the equations. Unless stated otherwise, model parameters used are as listed in Table I.

For the dynamics, we numerically solved the system dynamics with a model of deteriorating pulmonary function, modeled as a biphasic decay in Eq. [1] with:

[10] $\alpha\left( t \right)=\alpha_{\text{min}}+\left( \alpha_{\text{max}}-\alpha_{\text{min}} \right)\cdot\exp\left( -\frac{1}{4}\frac{t}{\alpha_{T}}-\frac{1}{2}\frac{t^{2}}{\alpha_{T}^{2}} \right)$

Here $\alpha\left( 0 \right)=\alpha_{\text{max}}=1.0$ is the absorbance in the healthy initial state, $\alpha\left( t\gg\alpha_{T} \right)=\alpha_{\text{min}}$ is the terminal absorbance, and $\alpha_{T}$ is the timescale for lung function to drop to approximately the midpoint. In all simulations we used $\alpha_{T}=5$ days and $\alpha_{min}=0.7$, indicating that the lung’s absorbance is only 70% of healthy lung capacity.

*4.2 Definition of performance goals.*

From the numerical solution of the model response to deteriorating pulmonary function (Eq. [10]), we define a time-scale for recovery by adapting the integral-time-square error (ITSE), a common metric for control system performance:

$$t_{ITSE}=\left[ \int_{0}^{\infty} t \max\left( \bar{pO_{2}}-pO_{2}\left( t \right),0 \right)^{2}dt \right]^{1/2}$$

For every parameter set considered, one can now define two performance goals: the response performance *s*=1/$t_{ITSE}$, and the constitutive over-production burden $\theta_{c}$ (Eq. [7]).

*4.3 Parameter scanning and response/burden* (*s*, $\theta_{c}$) *trade off analysis*

Here we describe the parameter scans shown in **Figs. S7E, 7E, 7F.** These figures plot the performance goals (*s*, $\theta_{c}$) for different parameter sets.

*4.3.1 Single-parameter scans* (**Fig. 7E**)

In **Fig. 7E**, performance goals are evaluated while varying one parameter at a time of the ranges shown in **Table II**.

**Table II:** Parameter ranges scanned.

| Parameter | Range |
| --- | --- |
| $\boldsymbol{\Delta}\boldsymbol{g}$ | 0.3-1.3/day |
| $\boldsymbol{K}_{\boldsymbol{a}}\boldsymbol{/}\boldsymbol{K}_{\boldsymbol{s}}$ | 0.05 - 20 |
| $\boldsymbol{n}_{\boldsymbol{a}}$ | 1 - 4 |
| $\boldsymbol{g}_{\boldsymbol{min}}$ | 0.3 – 1.3/day |
| $\boldsymbol{\tau}$ | 5-11 days |
| $\mathbf{S}_{\mathbf{MPP}}$ | $\left( 6.25-62.5 \right)\cdot{10}^{-5}$/day |

Using baseline parameters, the scan for $g_{min}$ was then used to construct **Fig. S7D**, showing the relationship between performance *s* and apoptosis rate $\left( 1-1/\theta_{c} \right).$

*4.3.2 Multi-parameter sampling* (**Fig. 7F, S7E**)

For **Fig. 7F, S7E**, we sampled 10^5^ parameters sets ${(g}_{min}, \Delta g,K_{a}/K_{s},n_{a})$ on a latin hypercube. Latin hypercube sampling was performed using subroutine *lhs* from python package pyDOE2 (v1.3.0). For each parameter value we then calculated the performance goals (*s*, $\theta_{c}$). The simulations were binned over (*s*, $\theta_{c}$) values, and the mean value of each parameter in each bin was plotted. The values of $g_{min}, \Delta g ,K_{a}/K_{s}$ given in the baseline table above are close to the pareto front.

**Table II:** Parameter ranges scanned.

| Parameter | Range |
| --- | --- |
| $\boldsymbol{\Delta}\boldsymbol{g}$ | 0.25-1.5/day |
| $\boldsymbol{K}_{\boldsymbol{a}}\boldsymbol{/}\boldsymbol{K}_{\boldsymbol{s}}$ | 0.05 - 20 |
| $\boldsymbol{n}_{\boldsymbol{a}}$ | 1 - 4 |
| $\boldsymbol{g}_{\boldsymbol{min}}$ | 0.3 – 1.0/day |

All remaining parameters have values as in **Table I**.

*4.4 Simulation of experimental injections under competing models of IL-17A action* (**Fig. 7G, S7F**)

The synergy plots shown in **Fig. 7G** and **Fig. S7F** provide a qualitative distinction between the two models. The simplifications made in model construction (see introduction and section 1 above) make this model unsuitable for *bona fide* data fitting and just one choice of parameters is sufficient to highlight qualitative differences between the hypotheses.

To generate the synergy plots shown in **Fig. 7G** and **Fig. S7F** we numerically solved the system dynamics (Eqs. [1-5]) as described in section 4.1 over the time interval *t=*[0,3] days and using the same baseline parameters (**Table 1)**, with the following modifications:

1. We held $\alpha\left( t \right)=\alpha_{max}=\mathrm{const}$ant.
2. To simulate Epo injection, we replaced Eq. [2] with the following:

$$E\left[ t \right]=\left\{ \begin{matrix} E_{0} \mathrm{for} t\leq0 \\ E_{0}+E_{inj.} for 0<t\leq3 \mathrm{days} \end{matrix} \right.$$

with $E_{inj.}=5K_{s}$

To simulate IL-17A injection, we acutely changed the model parameters as follows:

- 1. For Model 1 (direct control):
  $g_{min}=\left\{ \begin{matrix} 0.65/\mathrm{day} \mathrm{for} t\leq0 \\ 1/\mathrm{day} \mathrm{for} 0<t\leq3 \mathrm{days} \end{matrix} \right.$
  2. For Model 2 (Epo-sensitization)

$K_{a}/K_{s}=\left\{ \begin{matrix} 8 \mathrm{for} t\leq0 \\ 2 for 0<t\leq3 days \end{matrix} \right.$ (**Fig. 7G**)

or

$g_{max}-g_{min}=\left\{ \begin{matrix} 0.65/\mathrm{day} \mathrm{for} t\leq0 \\ 1.3/\mathrm{day} \mathrm{for} 0<t\leq3 \mathrm{days} \end{matrix} \right.$ (**Fig. S7F**)

For joint injections, both (2) and (3) were implemented. In each case, the dynamics were solved, and data points plotted correspond to values $\left( \left[ y_{k} \right]^{\left( E \right)}\left[ y_{k} \right]^{\left( I \right)}/\left( \left[ y_{k} \right]^{\left( 0 \right)} \right)^{2},\left[ y_{k} \right]^{\left( E+I \right)}/\left[ y_{k} \right]^{\left( 0 \right)} \right)$ for increasing values of *k* at time *t*=3 days post-injection.

**References**

1. Schirm, S. & Scholz, M. A biomathematical model of human erythropoiesis and iron metabolism. *Sci. Rep.* **10**, 8602 (2020).

2. Li, H. *et al.* Rate of Progression through a Continuum of Transit-Amplifying Progenitor Cell States Regulates Blood Cell Production. *Dev. Cell* **49**, 118-129.e7 (2019).

3. Koury, M. J. Tracking erythroid progenitor cells in times of need and times of plenty. *Exp. Hematol.* **44**, 653–663 (2016).

4. Koury, M. J. & Bondurant, M. C. The mechanism of erythropoietin action. *Am. J. Kidney Dis.* **18**, 20–23 (1991).

5. Ebert, B. L. & Bunn, H. F. Regulation of the erythropoietin gene. *Blood* **94**, 1864–1877 (1999).

6. Koury, M. J. & Bondurant, M. C. Erythropoietin retards DNA breakdown and prevents programmed death in erythroid progenitor cells. *Science* **248**, 378–381 (1990).

7. Socolovsky, M., Fallon, A. E., Wang, S., Brugnara, C. & Lodish, H. F. Fetal anemia and apoptosis of red cell progenitors in Stat5a-/-5b-/- mice: a direct role for Stat5 in Bcl-X(L) induction. *Cell* **98**, 181–191 (1999).

8. Dolznig, H. *et al.* Apoptosis Protection by the Epo Target Bcl-XL Allows Factor-Independent Differentiation of Primary Erythroblasts. *Curr. Biol.* **12**, 1076–1085 (2002).

9. Koury, M. J. & Bondurant, M. C. Maintenance by erythropoietin of viability and maturation of murine erythroid precursor cells. *J. Cell. Physiol.* **137**, 65–74 (1988).

10. Liu, Y. *et al.* Suppression of Fas-FasL coexpression by erythropoietin mediates erythroblast expansion during the erythropoietic stress response in vivo. *Blood* **108**, 123–133 (2006).

11. Peslak, S. A. *et al.* EPO-mediated expansion of late-stage erythroid progenitors in the bone marrow initiates recovery from sublethal radiation stress. *Blood* **120**, 2501–2511 (2012).

12. Myers, G. *et al.* Murine erythroid differentiation kinetics in vivo under normal and anemic stress conditions. *Blood Adv* **7**, 5727–5732 (2023).

13. Ganzoni, A., Hillman, R. S. & Finch, C. A. Maturation of the macroreticulocyte. *Br. J. Haematol.* **16**, 119–135 (1969).

14. Wiczling, P., Ait-Oudhia, S. & Krzyzanski, W. Flow cytometric analysis of reticulocyte maturation after erythropoietin administration in rats. *Cytometry A* **75**, 584–592 (2009).

15. Axelrad, A. A., McLeod, D. L., Shreeve, M. M. & Heath, D. S. Properties of cells that produce erythrocytic colonies in vitro. *Hemopoiesis in culture* 226 (1974).

16. Porpiglia, E., Hidalgo, D., Koulnis, M., Tzafriri, A. R. & Socolovsky, M. Stat5 signaling specifies basal versus stress erythropoietic responses through distinct binary and graded dynamic modalities. *PLoS Biol.* **10**, e1001383 (2012).

17. Peschle, C. *et al.* Identification and characterization of three classes of erythroid progenitors in human fetal liver. *Blood* **58**, 565–572 (1981).

18. Gregory, C. J. Erythropoietin sensitivity as a differentiation marker in the hemopoietic system: studies of three erythropoietic colony responses in culture. *J. Cell. Physiol.* **89**, 289–301 (1976).

19. Van Putten, L. M. The life span of red cells in the rat and the mouse as determined by labeling with DFP32 in vivo. *Blood* **13**, 789–794 (1958).
