# Supplementary material for: IL-17 stimulates erythropoiesis in vivo by amplifying the response of erythroid progenitors to erythropoietin
Source: PLoS Biol. 2025 Dec 11;23(12):e3003462. doi: 10.1371/journal.pbio.3003462 (PMC12697970; doi:10.1371/journal.pbio.3003462)
Supplement: S8 Table — (DOCX) [file pbio.3003462.s016.docx]

| Reference | Baseline IL-17A (plasma or serum of healthy controls, pg/ml) | IL-17A in Covid-19 or ARDS* (plasma or serum, pg/ml) | IL-17A in other pathology  (plasma or serum, pg/ml) | Fold change (pathology over baseline) | Diagnosis | Comments |
| --- | --- | --- | --- | --- | --- | --- |
| Sharif-Askari et al. ^1^ | 20 | 63 |  | 3 | Covid-19 |  |
| Mahallawi  et al. ^2^ | 2.5 | 190 |  | 76 | MERS-CoV | Numbers estimated from chart |
| Mostafa et al. ^3^ | 150 | 3000 (range: 750 -5400) |  | 20 | Covid-19 | a children's clinical study |
| Krawiec et al. ^4^ | 7 |  | 21 | 3 | Ulcerative colitis |  |
| Mikacenic et al. ^5^ | not done | 0.1 to 5000 |  |  | ARDS | Causes of ARDS include sepsis, pneumonia, trauma, aspiration |
| Ismail et al. ^6^ | medians: 50 -150  range: 20 -1000 |  | medians: 250 -500  range: 10- 4000 | 5 | Langerhans Cell Histiocytosis | Paper compares 3 ELISA approaches. Numbers estimated from chart |
| Boos et al. ^7^ | 0.46 ± 0.4 |  | 1.3 ± 3.0 | 3 | High altitude |  |
| Yuan et al. ^8^ |  |  |  | 12 to 50 | include Hepatitis C, Zika, MERS | meta-analysis of viral infections |

* ARDS, Acute Respiratory Distress Syndrome

1. Sharif-Askari, F.S., Sharif-Askari, N.S., Hafezi, S., Mdkhana, B., Alsayed, H.A.H., Ansari, A.W., Mahboub, B., Zakeri, A.M., Temsah, M.-H., Zahir, W., et al. (2022). Interleukin-17, a salivary biomarker for COVID-19 severity. PLOS ONE *17*, e0274841. 10.1371/journal.pone.0274841.

2. Mahallawi, W.H., Khabour, O.F., Zhang, Q., Makhdoum, H.M., and Suliman, B.A. (2018). MERS-CoV infection in humans is associated with a pro-inflammatory Th1 and Th17 cytokine profile. Cytokine *104*, 8-13. <https://doi.org/10.1016/j.cyto.2018.01.025>.

3. Ahmed Mostafa, G., Mohamed Ibrahim, H., Al Sayed Shehab, A., Mohamed Magdy, S., AboAbdoun Soliman, N., and Fathy El-Sherif, D. (2022). Up-regulated serum levels of interleukin (IL)-17A and IL-22 in Egyptian pediatric patients with COVID-19 and MIS-C: Relation to the disease outcome. Cytokine *154*, 155870. <https://doi.org/10.1016/j.cyto.2022.155870>.

4. Krawiec, P., and Pac-Kożuchowska, E. (2020). Serum interleukin 17A and interleukin 17F in children with inflammatory bowel disease. Scientific Reports *10*, 12617. 10.1038/s41598-020-69567-x.

5. Mikacenic, C., Hansen, E.E., Radella, F., Gharib, S.A., Stapleton, R.D., and Wurfel, M.M. (2016). Interleukin-17A Is Associated With Alveolar Inflammation and Poor Outcomes in Acute Respiratory Distress Syndrome. Crit Care Med *44*, 496-502. 10.1097/ccm.0000000000001409.

6. Ismail, M.B., Åkefeldt, S.O., Lourda, M., Gavhed, D., Gayet, R., Aricò, M., Henter, J.-I., Delprat, C., and Valentin, H. (2020). Comparison of three different ELISAs for the detection of recombinant, native and plasma IL-17A. Methods X *7*. 10.1016/j.mex.2020.100997.

7. Boos, C.J., Woods, D.R., Varias, A., Biscocho, S., Heseltine, P., and Mellor, A.J. (2015). High Altitude and Acute Mountain Sickness and Changes in Circulating Endothelin-1, Interleukin-6, and Interleukin-17a. High Altitude Medicine & Biology *17*, 25-31. 10.1089/ham.2015.0098.

8. Yuan, S., Jiang, S.-C., Zhang, Z.-W., Fu, Y.-F., Hu, J., and Li, Z.-L. (2021). Quantification of Cytokine Storms During Virus Infections. Frontiers in immunology *12*. 10.3389/fimmu.2021.659419.
